# The role of the water contact layer on hydration and transport at solid/liquid interfaces

**DOI:** 10.1073/pnas.2407877121

**Published:** 2024-09-11

**Authors:** J. Gäding, V. Della Balda, J. Lan, J. Konrad, M. Iannuzzi, R. H. Meißner, G. Tocci

**Affiliations:** ^a^Institute of Soft Matter Modeling, Hamburg University of Technology, Hamburg 21073, Germany; ^b^Institute of Surface Science, Department of Atomistic Corrosion Informatics, Helmholtz-Zentrum Hereon, Geesthacht 21502, Germany; ^c^Department of Chemistry, University of Zurich, Zurich 8057, Switzerland; ^d^Department of Chemistry, New York University, New York, NY 10003; ^e^Department of Chemistry, Simons Center for Computational Physical Chemistry at New York University, New York, NY 10003

**Keywords:** molecular dynamics, aqueous interfaces, machine learning, nanofluidics

## Abstract

Understanding the structure in the nanoscopic region of water that is in direct contact with solid surfaces, so-called contact layer, is key to quantifying macroscopic properties that are of interest to e.g. catalysis, ice nucleation, nanofluidics, gas adsorption, and sensing. We explore the structure of the water contact layer on various technologically relevant solid surfaces, namely graphene, MoS_2_, Au(111), Au(100), Pt(111), and Pt(100), which have been previously hampered by time and length scale limitations of ab initio approaches or force field inaccuracies, by means of molecular dynamics simulations based on ab initio machine learning potentials built using an active learning scheme. Our results reveal that the in-plane intermolecular correlations of the water contact layer vary greatly among different systems: Whereas the contact layer on graphene and on Au(111) is predominantly homogeneous and isotropic, it is inhomogeneous and anisotropic on MoS_2_, on Au(100), and on the Pt surfaces, where it additionally forms two distinct sublayers. We apply hydrodynamics and the theory of the hydrophobic effect, to relate the energy corrugation and the characteristic length-scales of the contact layer with wetting, slippage, the hydration of small hydrophobic solutes and diffusio-osmotic transport. Thus, this work provides a microscopic picture of the water contact layer and links it to macroscopic properties of liquid/solid interfaces that are measured experimentally and that are relevant to wetting, hydrophobic solvation, nanofluidics, and osmotic transport.

## Significance

The advancement of energy production, catalysis, and chemical processes is imperative to address contemporary challenges, notably climate change. To achieve this, a profound comprehension of the intricate interplay between microscopic material structures and dynamic properties, and their consequential impact on macroscopic behavior, is crucial. In this pursuit, our study employs purpose-made state-of-the-art machine learning techniques at the atomic level to explore six technologically significant water/solid interfaces with near ab initio accuracy. Through extensive dynamic simulations, this work unveils distinctive static and dynamic properties of the water contact layer, establishing crucial correlations with hydration, transport, and wetting characteristics.

Water can form a remarkably rich variety of structures when in contact with solid surfaces depending on, for example, surface morphology, chemical composition, crystal structure, and humidity. Due to the intimate relation between structure and property of materials and interfaces, understanding the microscopic structure of water at solid surfaces is, therefore, central to achieve insights into technologically and scientifically relevant processes. Specifically, a large body of work indicates that the water layer that is in direct contact with the surface plays a crucial role in heterogeneous ice nucleation (1), in nanofluidic slippage (2–5), in the evolution of hydrogen at metal electrodes (6), in wetting (7–9), in energy conversion, and its storage (10–12). The interfacial properties of water in direct contact with the underlying substrate can be remarkably different, depending on the surface topography and chemical composition. For example, so-called “nano-impact” electrochemistry experiments of aqueous electrolytes at the interface with metal nanoparticles revealed a larger electric double-layer capacitance on Pt nanoparticles compared to Au nanoparticles due to the different structure of the water contact layer on the two metal substrates, as illuminated by molecular simulations (12). Even in the case of a chemically pure substrate, exposing water to different surface orientations can have a strong impact on the resulting water structure and dynamics, as well as on wetting and reactivity (7). Additionally, experimental evidence of the richness of water structures formed on defect-free surfaces has been obtained from surface-science studies performed in ultrahigh vacuum conditions and at cryogenic temperatures (13). Despite much progress, probing the structure of the contact layer of water in its liquid phase is arguably more challenging, as many of the techniques that have successfully been used to characterize clean surfaces under ultrahigh vacuum conditions are hampered by the presence of high vapor pressure of aqueous solutions at ambient temperatures, and of possible contaminants from the bulk (14, 15).

Molecular dynamics (MD) simulations, especially when exploring the intricate nature of water/solid interfaces, have provided invaluable insights into structure and dynamics. Nevertheless, these simulations come with inherent limitations as highlighted, for instance, in ref. 15. First, creating accurate empirical force fields across multiple aqueous systems proves to be a challenging task. Second, the scope of time-scales and system sizes that ab initio molecular dynamics simulations can cover might provide an insufficient sampling of the complex interfacial properties. Such limitations hinder the in-depth study of interfacial water, especially when considering varied chemical compositions and crystal orientations. Addressing these challenges, Committee Neural Network Potentials (C-NNPs), which consist of multiple high-dimensional neural network potentials (HDNNPs), offer a promising solution. These potentials, trained on ab initio molecular dynamics trajectories, can achieve an accuracy comparable to that of the underlying ab initio simulations at a fraction of the computational cost (16–18). This underscores the pivotal role of systematic investigations on model-substrates using MD powered by HDNNPs. Such studies serve as a bridge, connecting the microscopic structure of water on solid surfaces to experimentally observable quantities of significant technological implications.

Building on this premise, our work addresses two core questions, explored via comprehensive MD simulations anchored by the accuracy and cost-efficiency of HDNNPs trained on ab initio data: i) How are the microscopic details of the water contact layer influenced by the different chemical nature and crystal orientation of the substrate? ii) How does the microscopic structure of the contact layer in turn influence macroscopic properties that are relevant to wetting, to hydrophobic hydration, to slippage, and to diffusio-osmotic transport? To answer these questions, we investigate water at the interface with metal surfaces that are of special interest in electrochemistry, such Au(100), Au(111), Pt(100), and Pt(111) (13), and with two-dimensional materials that are relevant for energy storage and osmotic energy conversion, such as graphene and MoS_2_ (19–21). We find that the molecular structure of the contact layer, exemplified by the water pair correlation function in the direction parallel to the surface, varies substantially on each substrate. Whereas the pair correlation function at the aqueous graphene interface is short-range isotropic and long-range homogeneous, that on the metal surfaces, especially Pt(100), reveals a strong short-range anisotropy and long-range inhomogeneity. Additionally, the contact layer on both platinum surfaces forms sublayers with chemisorbed water molecules. The hydration of the metal surfaces and of the two-dimensional materials under investigation are intimately connected to the microscopic structure of the contact layer, as are the binding and transport of molecular-sized hydrophobic solutes and the nanofluidic slip. Specifically, we shed light on the hydrophobicity of the materials investigated and connect it to a length-scale that is characteristic of the solid/liquid interface. Further, we show that hydration of hydrophobic solutes is largely determined by the height fluctuations of the water contact layer and by the width of a vacuum-like region between the surface and the contact layer. Finally, we illustrate that solid/liquid friction is strongly influenced by the in-plane corrugation of the contact layer and that this, in turn, has a dramatic impact on the diffusio-osmotic transport of hydrophobic solutes.

## Results and Discussion

Here, we present the main results of this work, structured into three interconnected sections. We first discuss the water density profiles, the in-plane intermolecular correlations, and the corrugation of the water layer in contact with the two-dimensional materials and with the metal surfaces, providing a detailed perspective on the microscopic structure of the water contact layer. Next, we connect these results with large-scale hydration, i.e. wetting, and the hydration of small hydrophobes. In the final section, we explore the relationship with solid/liquid friction and diffusio-osmotic transport at the interface.

### Microscopic Structure of the Water Contact Layer.

**Fig. 1.**
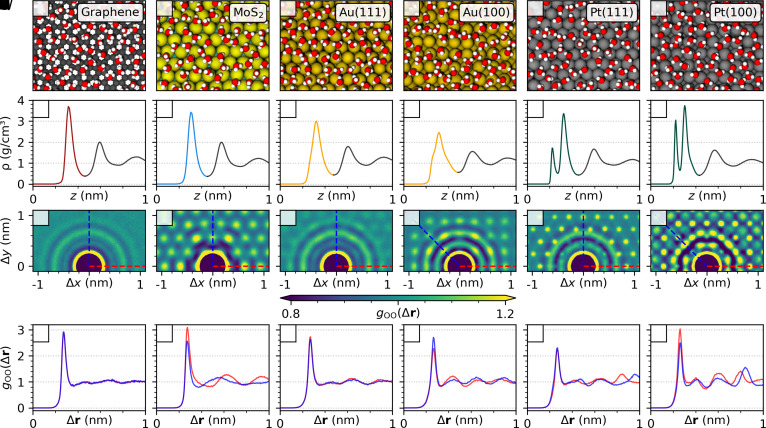
(*A*–*F*) Snapshots of the water contact layer of the aqueous interfaces on graphene, MoS_2_, Au(111), Au(100), Pt(111), and Pt(100). (*G*–*L*) Water density profiles with respect to the instantaneous solid interface. The colored part of the respective density profile corresponds to the water contact layer. (*M*–*R*) Two-dimensional representation of the oxygen pair correlations $g_{\mathrm{OO}}\left(\Delta r\right)$ within the water contact layer in the direction parallel to the surface. High symmetry axes are shown in blue, respectively red dotted lines. (*S*–*X*) One-dimensional representation of $g_{\mathrm{OO}}\left(\Delta r\right)$ along the indicated high symmetry axis.

**Fig. 2.**
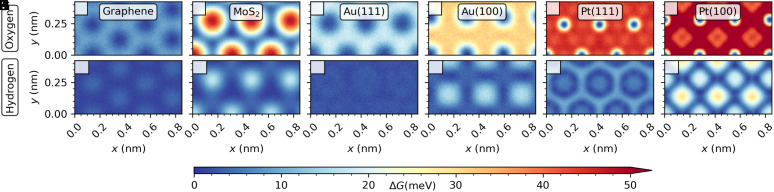
Free energy landscape of water within the contact layer on graphene, MoS_2_, Au(100), Au(111), Pt(100), and Pt(111) felt by the (*A*–*F*) oxygen atoms and (*G*–*L*) hydrogen atoms, respectively. Each unit cell of the free energy surface is oriented so that its origin corresponds to the center of a substrate atom and is therefore located at x=0.0 nm and y=0.0 nm.

The snapshots in Fig. 1 (top row) show representative structures of the water contact layer at the solid/liquid interfaces under investigation. Through the analysis of the density profiles (second row), of the two-dimensional pair correlation functions (2D-PCFs) and their line-profiles (1D-PCFs) (third and fourth row), we shed light on the structure and ordering within the water contact layer. The water contact layer on graphene presents a pronounced density peak reaching about 3.8 g/cm^3^ at a height of about 3.5 Å, which is characteristic of water physisorption. Despite the pronounced structuring in the direction perpendicular to the surface, the in-plane structure of the water contact layer on graphene displays features characteristic of an isotropic fluid in the short-range, as evinced by the circular pattern of the first three solvation shells in the $g_{\;\text{OO}}\left(\Delta r\right)$ in Fig. 1*M*. The one-dimensional cuts along the zig-zag and armchair direction (Fig. 1*S*) are indistinguishable, further supporting this observation. Additionally, at long distances, the 2D-PCF and the 1D-PCF approximately tend to the ideal gas limit, meaning that the contact layer is homogeneous, and no pronounced long-range ordering is observed. The homogeneous and isotropic structure of the contact layer on graphene is further confirmed by a particularly small corrugation in the two-dimensional free energy surface (FES) compared to thermal energy (see e.g. Fig. 2*A*), where thermal energy at room temperature is $k_{B}T\approx 26$ meV.

On MoS_2_ the water density (Fig. 1*H*) perpendicular to the surface shows, besides a slightly less pronounced density peak of 3.5 g/cm^3^, a very similar structure as on graphene. However, the 2D- and 1D-PCF show that the water contact layer is akin to a short-range anisotropic and long-range inhomogeneous fluid. In particular, the first solvation shell shows an anisotropic distribution with different peak heights in the 1D-PCF (Fig. 1*T*) along the two orthogonal symmetry axes. Moreover, the 2D-PCF shows that the circular second and third solvation shells are suppressed, so that the distribution is increasingly dictated by the hexagonal pattern of the underlying MoS_2_ substrate. The preferred adsorption sites are located in the valleys between the sulfur atoms, as evident by the FES (see e.g. Fig. 2*B*). As the distance between these preferred binding sites is closer to the optimal water–water distance of 3.2 Å along the [100] lattice (x-direction), the density peaks are increased along this axis. Between the solvation shells, there are regions of strongly reduced density due to the significant suppression of water adsorption on the Mo sites.

On Au(111), the density profile in the contact layer is broader compared to graphene and MoS_2_ and less pronounced, as it presents a shoulder close to the surface and reaches a value of about 3.0 g/cm^3^ (Fig. 1*I*). The short-range in-plane structure of the contact layer is predominantly isotropic, as seen in the circular pattern of the 2D-PCF up to the third solvation shells. However, water preferably adsorbs on the top site on Au(111) and the corrugation of the water contact layer is estimated to be approximately 20 meV (Fig. 2*C*), just below the thermal energy regime. Thus, long-range ordering, characteristic of an inhomogeneous fluid (22), is observed in the 2D-PCF approximately beyond the third solvation shell, with the emergence of a hexagonal pattern, reflecting the symmetry of the underlying surface lattice.

On Au(100) the density peak of the contact layer decreases further, and presents a more pronounced shoulder compared to Au(111) (Fig. 1*J*), indicating that water adsorption is stronger than on previous surfaces. The 2D- and 1D-PCF reveal that the water contact layer on Au(100) is characteristic of a short-range anisotropic and long-range inhomogeneous fluid. The first solvation shell showcases an anisotropic distribution with varying peak heights in the 1D-PCF (Fig. 1*V*). Furthermore, the 2D-PCF reveals a distinctive pattern characterized by regions of increased density in the second and third solvation shells along high symmetry axes. The observed long-range pattern follows the square lattice structure of the underlying Au(100) substrate and persists throughout the entire contact layer. Notably, the oxygen atoms preferably adsorb on the top site, and the corrugation in the FES is close to the thermal energy (see the FES in Fig. 2*D*).

The water density profile on Pt(111), shown in Fig. 1*K*, reveals a separation of the contact layer into two sublayers, as evident by two distinct peaks, with one less pronounced peak in proximity of the surface and a denser second peak. In particular, the first peak appears at a height of about 2.0 Å from the surface and it is a sign of chemisorption, as opposed to the previously discussed materials, where water adsorbs less strongly. Although water adsorbs strongly on Pt(111), the short-range in-plane structure of the contact layer is interestingly isotropic, as demonstrated by the circular ring pattern of the first solvation shell in the 2D-PCF and in the overlapping peaks along the high symmetry axes seen in the 1D-PCF (Fig. 1 *Q* and *W*). However, beyond the third solvation shell, a hexagonal pattern is observed in the 2D-PCF, indicating long-range inhomogeneity of the water contact layer that can be attributed to the increased energy corrugation of approximately 40 meV in the FES (*cf.* Fig. 2*E*), which is now markedly larger than the thermal energy.

Similar to Pt(111), the contact layer on Pt(100) exhibits a separation into two sublayers, with higher-density maxima compared to Pt(111) as a sign of yet stronger interaction with the surface (Fig. 1*L*). A detailed analysis of the individual sublayers of Pt(111) and Pt(100) is given in *SI Appendix*, section S3. Notably, the emergence of two sublayers on Pt(100) and on Pt(111) has been observed in recent MD studies using first-principles methods (23–27) and many-body empirical potentials (28). According to the 2D-PCF shown in Fig. 1*R* its in-plane structure exhibits the most pronounced signatures of short-range anisotropy and long-range inhomogeneity. The anisotropy of the first solvation shell is visible in the 1D-PCF (Fig. 1*X*), where the peak’s magnitude changes depending on the orientation. Long-range correlations, approximately beyond the third solvation shell also are seen in the 2D-PCF and form a square lattice according to the symmetry of the underlying substrate. The strong attraction of water to the surface leads to the highest corrugation in the FES among the studied interfaces, of approximately 65 meV, with water preferably sitting on the top site. Additionally, there is an intermediate region in the 2D-PCF corresponding to the second and third solvation shells, where the in-plane structure appears to be frustrated due to a competition between strong water–water and water–substrate interactions.

To summarize, the short-range and long-range ordering described by the in-plane intermolecular correlations of the contact layer are dictated by the underlying symmetry of the substrate and by the strength of the water/surface interactions that can be quantified by the corrugation of the FES on each material. Since graphene has a large number of adsorption sites per surface area and each site interacts weakly with water compared to the other materials, the intermolecular correlations reveal a short-range isotropic and long-range homogeneous contact layer. On close-packed metal surfaces with hexagonal symmetry [Au(111) and Pt(111)], the short-range structure characterized by the 2D-PCF is isotropic, whereas it is anisotropic on MoS_2_ and open surfaces with square symmetry [Au(100) and Pt(100)]. Approximately beyond the third solvation shell, water/surface interactions may lead to long-range ordering, with the 2D-PCF adapting to the symmetry of the substrate. The level of corrugation of the FES, compared to thermal energy, determines the amplitude of such long-range inhomogeneities observed in the 2D-PCF of the contact layer. This indicates that increasingly larger corrugation in the FES gives rise to the following progression, where the magnitude in the long-range oscillations also increases with the progression: graphene < Au(111) < Au(100) < MoS_2_< Pt(111) < Pt(100).

### Hydrophobes’ Adsorption and Connection to Wetting.

**Fig. 3.**
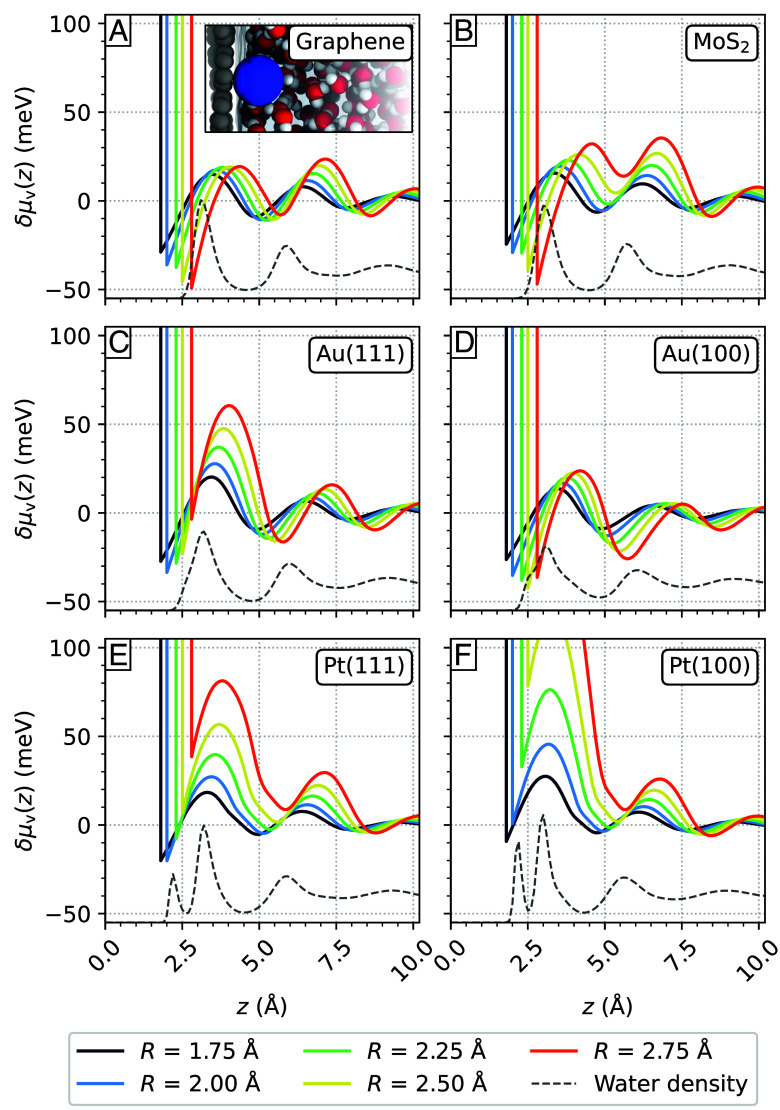
Excess solvation free energy profiles $\delta \mu_{v}\left(z\right)$ for hydrophobes with radii R ranging from 1.75 Å to 2.75 Å at the aqueous (*A*) graphene, (*B*) MoS_2_, (*C*) Au(111), (*D*) Au(100), (*E*) Pt(111), and (*F*) Pt(100) interface as a function of the distance between the hydrophobes and the surface z. The water density profile at each interface is shown qualitatively by a gray dotted line. The *Inset* in (*A*) illustrates a hydrophobe (blue) with a radius of 2.50 Å stabilized within a cavity at the graphene interface in close proximity to the solid surface.

In this section, we establish a connection between the microscopic structure of the contact layer and the free energy of adsorption of small hydrophobic solutes. We also relate the height fluctuations of the water contact layer to surface hydration and explicitly extract the water contact angle from the size dependence of the free energy of adsorption of the hydrophobic solutes. The free energy of solvation δμ(z) of hydrophobes as a function of height from the surface is shown in Fig. 3 for the system investigated, where small hydrophobic solutes are modeled as spherical cavities with radii below 0.3 nm. In bulk water, the solvation of hydrophobes with radii below approximately 0.7 nm is associated with a distortion of the water hydrogen-bond network, thus providing an entropic contribution to the free energy of solvation, whereas larger radii are associated with an enthalpic term due to the breaking of hydrogen bonds (29, 30). At the interface with a solid however, there is an interplay between several effects, and the transition between entropy-driven and enthalpy-driven solvation may occur for smaller solute radii compared to hydrophobic hydration in the bulk liquid (31). Besides the distortion and breaking of hydrogen bonds at the aqueous interface, water density oscillations can lead to the stabilization of hydrophobes in regions with a lower density than in the bulk, and vice versa for regions where there are pronounced density peaks. Further, the presence of a vacuum-like region between 0.2 nm and 0.3 nm that separates the surface from the water contact layer may lead to the stabilization of hydrophobes in this region (32).

At the graphene/water and at the MoS_2_/water interfaces (Fig. 3 *A* and *B*), hydrophobe hydration is more stable compared to bulk water, and the global minima in δμ(z) are close to the surface, at a height z between 0.2 nm and 0.3 nm. In this region, the adsorption of hydrophobes is further stabilized for hydrophobes of increasingly larger radii, with the free energy going from about −25 meV to −50 meV for particles with radii of R=0.175 nm and R=0.275 nm, respectively. This phenomenon can be attributed to the presence of a thin vacuum region between the surface and the first water density peak, which is about 0.3nm-wide. On graphene, solvation of hydrophobes near the transition layer is slightly more stable than in the bulk, whereas on MoS_2_ only the smallest particles with R≤0.2 nm feature a slight stabilization in the transition region with δμ(z)≈−9 meV. Although the graphene/water and the MoS_2_/water interface present similar density profiles, substrate-induced structuring in the direction parallel to the surface observed on MoS_2_, might lead to a decreased stabilization of hydrophobes in the transition region at this interface. Beyond the second free energy minimum, decaying oscillations in δμ(z) are observed with an amplitude of oscillations that increases with particles’ size.

Between the surface and the contact layer, the size-dependent hydration of hydrophobes at the Au(111)/water interface (Fig. 3*C*) is markedly different from the previous 2D materials. Within this region, the free energy minima decrease in stability as particle size increases: While for the smallest radii, solvation is stable and close to thermal energy, it is almost identical to the solvation in the bulk liquid for the largest considered particles. The first water density peak on Au(111) exhibits a shoulder in the direction of the surface and the water density profile extends to shorter distances compared to the density profiles on the two-dimensional materials, thus explaining the destabilization of hydrophobes’ adsorption near the surface as a function of particle size. Near the transition layer, solvation of hydrophobes is slightly more stable than in the bulk, due to water depletion between the contact layer and the transition layer, although the observed free energy minima do not go beyond −20 meV.

Hydrophobes’ solvation is, on Au(100), most stable close to the surface for all considered particle radii and nearly constant with their size, being in a range between −40 meV and −30 meV. Compared to Au(111) the first water density peak presents a wider shoulder in the direction of the surface which leads to a destabilizing effect. However, the density of surface atoms on Au(100) is smaller than on Au(111), ultimately leading to an increased stabilization of hydrophobes on Au(100).

Finally on Pt(111) and Pt(100), the adsorption of hydrophobes near the surface is less stable relative to the bulk as the particle size increases (Fig. 3 *E* and *F*). Only on Pt(111) the adsorption for the smallest particle considered, with R=0.20 nm, is about 20 meV more stable than in the bulk. Whereas for larger particle sizes solvation near the surface is either near degenerate to or less stable than in the bulk. The water contact layer is significantly more structured on Pt surfaces compared to Au surfaces due to water chemisorption, as seen in the water density profiles, ultimately leading to a destabilizing effect on hydrophobic hydration.

**Fig. 4.**
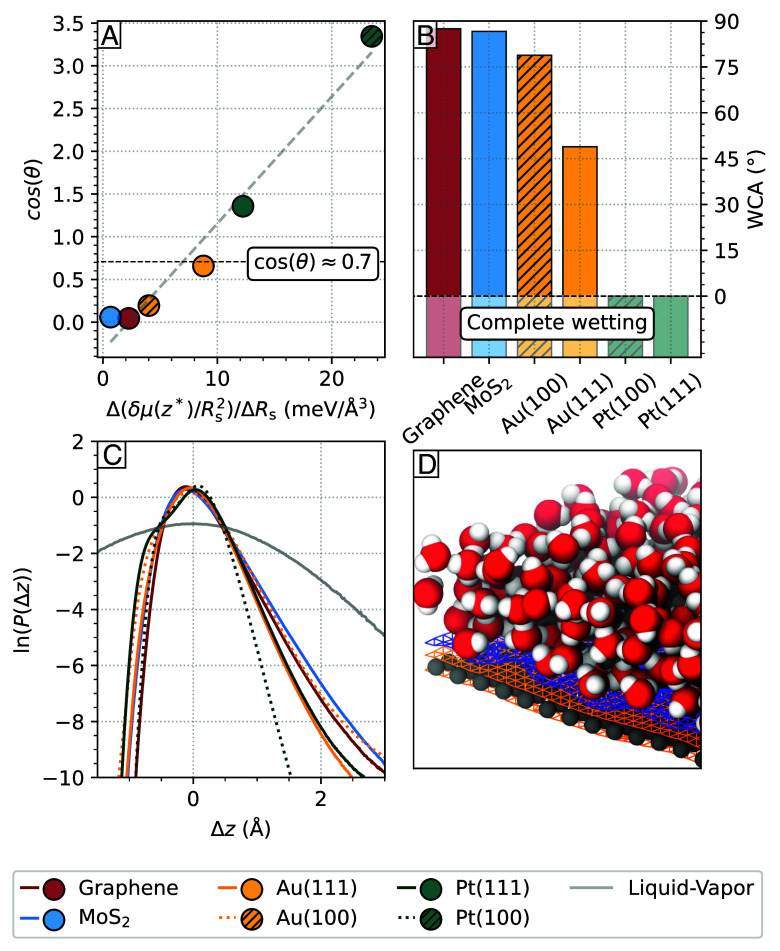
(*A*) The theoretical cosine of the water contact angle cos(θ) as a function of the progression of the cavity surface area normalized solvation free energy $\Delta (\mu \left(z^{*}\right)/R^{2}$ with the hydrophobes radius ΔR. The attraction–repulsion boundary for hydrophobic solutes at cos(θ)=0.7 is shown as a dashed line (33). (*B*) Water contact angle of the substrates; for completely wetting substrates (cos(θ)>1), there is naturally a zero contact angle. (*C*) Logarithmic probability distributions of height fluctuations ln(P(Δz)) of the instantaneous dividing interface relative to its average height $\bar{h}$. Negative values correspond to height fluctuations in the direction of the substrate, while positive values correspond to fluctuations in the direction of the liquid phase. (*D*) Snapshot of the water/graphene interface with the instantaneous liquid and solid surfaces shown in blue and orange.

Following the approach outlined by Godawat et al. (33), the water contact angle (WCA) θ is calculated based on the progression of excess solvation free energy and the corresponding bulk solvation energy. Fig. 4*A* depicts the calculated theoretical cosine value of the WCA, cos(θ), as a function of the change in solvation free energy normalized by the surface area of the cavity, $\left(\mu \left(z\right)/R^{2}\right)$, with respect to the hydrophobe radius R. The apparent linear relationship indicates that the cubic dependence between free adsorption energy and particle radius, as described by Chandler et al. (30) in bulk and confirmed for particles with R≤3 nm at hydrophilic interfaces (34), is further applicable for hydrophobic substrate interfaces.

Graphene, MoS_2_, and Au(100) demonstrate nonwetting behavior, indicated by cos(θ)<0.7 as shown in Fig. 4*A*. Au(111) exhibits neutral adsorption of a hydrophobic particle, presenting a borderline behavior between hydrophobic and hydrophilic with cos(θ)=0.7. Both platinum substrates, Pt(111) and Pt(100), display complete wetting. A consistent trend of hydrophobe stabilization and contact angle dependence with increasing size is observed across all substrates. Nonwetting substrates, particularly MoS_2_, show enhanced hydrophobe stabilization in the interfacial region of the contact layer with increasing size. Conversely, the metallic, nonwetting surface exhibits reduced hydrophobe stabilization or increased depletion with growing hydrophobe size in its interfacial region.

Fig. 4*B* illustrates the WCA in angular dimension. For graphene, characterized as the least wetting substrate, a calculated WCA of approximately $\theta \approx 87^{{}^{\circ}}$ is reported. Experimental and theoretical studies have reported varying WCAs for water on graphene, ranging from $42^{{}^{\circ}}$ (35) to $90^{{}^{\circ}}$ (36–38), or even as high as $127^{{}^{\circ}}$ (36), consistent with its hydrophobic nature as observed in our case. MoS_2_ exhibits a calculated WCA of $86^{{}^{\circ}}$, consistent with experimentally determined values for bulk configurations (39), although its monolayer configuration may yield smaller experimental WCAs. Comparisons of theoretically determined contact angles with experimentally observed values remain challenging due to finite size effects, ideal substrate structure and purity, as well as experimental setup variations. Comparisons with literature for metallic substrates are even more complex due to surface reconstructions in experiments, particularly for the unstable Au(100) surface (40, 41), or the absence of available values. Nevertheless, these substrates are generally associated with complete wetting behavior (42, 43).

Regarding wettability, the dynamic behavior of the instantaneous dividing interface on a substrate, beyond the static adsorption of hydrophobes, plays an insightful role in understanding wettability (44). The probability distribution of the height of this interface between the substrate and the water contact layer, denoted as P(Δz) (Eq. **4**), establishes a link between the microscopic dynamics of the contact layer and a macroscopic estimation of substrate hydrophobicity. Height fluctuations are influenced by solid/liquid interactions and compete with entropy-driven capillary wave fluctuations, usually following a Gaussian distribution (45–47). Weakly interacting yet hydrophobic surfaces limit the movement of the instantaneous liquid interface, restricting fluctuations toward the substrate. However, fluctuations are not suppressed in the direction of the liquid phase, resulting in pronounced non-Gaussian tails in the probability distribution (44). Stronger interacting hydrophilic surfaces further reduce fluctuations in the direction of the liquid phase, resulting in a narrower distribution with less pronounced tails. Fig. 4*C* illustrates the probability distribution lnP(Δz) of the instantaneous dividing interface for the considered systems.

Overall, there is a consistent correlation between increased wettability, expressed by the water contact angle, and suppressed surface fluctuations. Less hydrophilic surfaces like MoS_2_, graphene, and Au(100) exhibit increased fluctuations in their instantaneous dividing interface, shown by the broader distribution with general increased non-Gaussian tailing in lnP(Δz). Although Au(100) has the broadest distribution, it is not the least hydrophilic substrate. The broad distribution in Au(100) results from the widening of the contact layer, as indicated by the discussed shoulder in its density profile.

Comparing both Au substrates, Au(100) displays larger fluctuations than Au(111), underscoring the role of the surface density of adsorption sites in determining hydrophobicity. Despite stronger water–solid interactions on Au(100) compared to Au(111), the fewer adsorption sites on Au(100) lead to weaker pinning of the contact layer, even though each interaction is more robust, and the in-plane structure of the contact layer is more ordered.

The height distribution of the water contact layer on Pt(111) exhibits a tail in the direction of the bulk similar to that seen on Au(111), despite Pt(111) having stronger water–solid interactions. Unlike the previous interfaces, Pt(111) presents a pronounced shoulder for Δz<0, indicating the separation of the contact layer into two distinct sublayers. Finally, Pt(100) displays the most suppressed height fluctuations in the instantaneous dividing interface, evident in its smallest tails. The increased water density in the first sublayer compared to Pt(111) results in a more pronounced shoulder in the height distribution on Pt(100).

Combining the adsorption of hydrophobes and the fluctuations in the instantaneous dividing interface reveals two opposite scenarios: In the more hydrophobic case, the water contact layer is weakly bound to substrates with strong fluctuations of the instantaneous dividing interface, as well as an approximate 3Å thick vacuum-like region between the surface and the contact layer. In this scenario, exemplified by the aqueous graphene interface, larger hydrophobic solutes are increasingly more stable near the surface relative to the bulk. In the opposite, more hydrophilic scenario, where water is adsorbed more strongly, such as on Pt(100), the hydration of hydrophobes is not stable compared to the bulk liquid due to suppressed height fluctuations of the instantaneous water interface and a reduced vacuum-like region between the contact layer and the surface. This interpretation is consistent with the role of the hydrogen bonding network on the hydration of hydrophobes at the interface, where in-plane hydrogen bonding tends to stabilize hydrophobes, whereas interlayer hydrogen bonding has a destabilizing effect (31).

### Water Slippage and Amplification of Diffusio-Osmotic Transport.

**Fig. 5.**
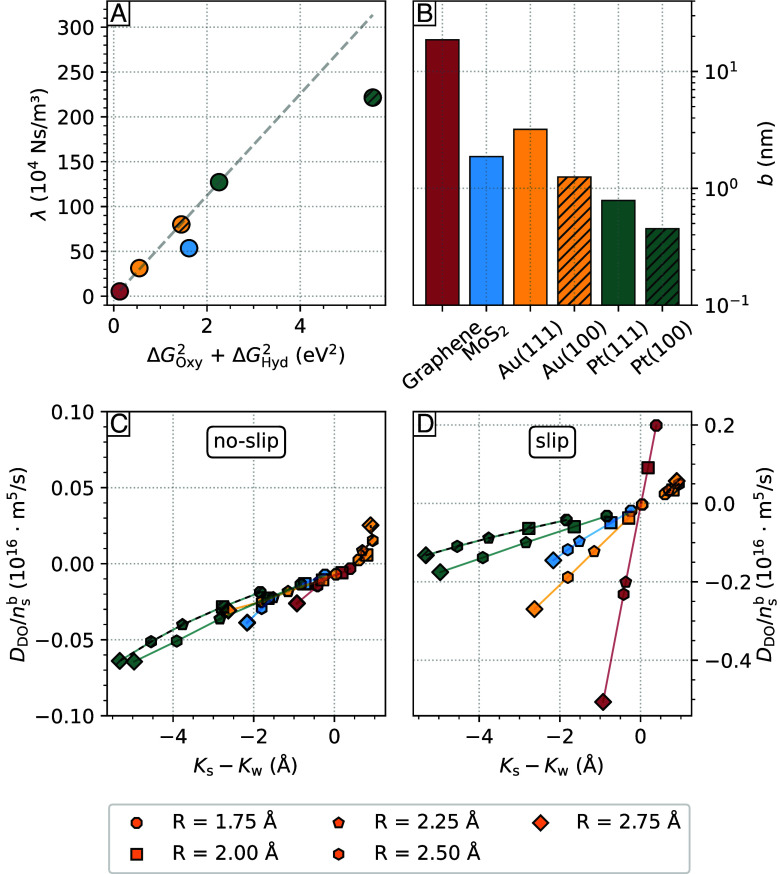
(*A*) The relationship between the friction coefficient λ and the squared corrugation in the free energy surface of water within the contact layer (denoted as ΔG in Fig. 2). (*B*) The slip length b of water at 330 K on graphene, MoS_2_, Au(111), Au(100), Pt(111), and Pt(100), presented in a logarithmic scale. The diffusio-osmotic mobility $D_{\mathrm{DO}}$ of a hydrophobic species normalized by the bulk concentration of $n_{s}^{b}$ illustrated as a function of the difference in the hydrophobe adsorption length and the water adsorption length $\left(K_{s}-K_{w}\right)$ for (*C*) no-slip and (*D*) slip conditions. The coloring follows the scheme in Fig. 4.

In addition to the distinct wetting behavior of the substrates, the observed intermolecular correlations and ordering of the water contact layer significantly impact transport properties at the solid/liquid interface. One crucial property influenced by these factors is the solid/liquid friction coefficient, denoted as λ, along with the slip length, represented by b. Indeed, solid/liquid friction has a major structural origin, which can be estimated by the corrugation of the FES in the contact layer (ΔG) (48–52). Specifically, a scaling relation has been identified (53) in the study of solid/liquid friction in C- and BN-nanotubes (53) by considering the FES of the oxygen ($\Delta G_{\mathrm{Oxy}}$) and of the hydrogen ($\Delta G_{\mathrm{Hyd}}$) atoms belonging to the water molecules in the contact layer, namely $\lambda ∼\Delta G_{\mathrm{Oxy}}^{2}+\Delta G_{\mathrm{Hyd}}^{2}$. In Fig. 5*A*, the friction coefficient for each material is plotted as a function of the sum of $\Delta G_{\mathrm{Oxy}}^{2}$ and $\Delta G_{\mathrm{Hyd}}^{2}$. Graphene has the lowest friction coefficient among all materials ($\lambda \approx 6\times 10^{4}$ Ns/m^3^) due to a very smooth free energy surface (48, 50, 53). The scaling relation $\Delta G_{\mathrm{Oxy}}^{2}$ and $\Delta G_{\mathrm{Hyd}}^{2}$ remains valid across all the materials investigated, except for Pt(100) and MoS_2_, where a slight deviation is observed. On Pt(100), an explanation is given by the presence of chemisorbed water molecules in the first sublayer leading to the formation of a stagnant layer. On MoS_2_, the decrease in friction coefficient is attributed to the existence of uninterrupted paths of low corrugation within the FES. This phenomenon is comparable to the observed reduction of friction in carbon nanotubes with varying chiralities (54). The bar chart in Fig. 5*B* shows the slip length b for each material, with b being above the characteristic size of 1 nm for all systems (55), with the exception of Pt(100) and Pt(111), where water adsorbs too strongly to give rise to significant water slippage. Overall, this illustrates the importance of adopting the partial-slip boundary condition when computing flow properties at solid/liquid interfaces, even on metal surfaces such as gold. Note that quantum friction may also be nonnegligible on gold surfaces due to the presence of surface plasmons (56, 57). Considering that the (nonquantum) slip length obtained here on Au(111) is already large enough to be measured in flow experiments (2), Au(111) may indeed represent an interesting test-case to probe quantum-friction at solid/liquid interfaces. We also note that in a pioneering work based on extensive MD simulations of liquid/solid interfaces, a quasi-universal relationship between contact angle and slip length has been established (58). However, according to an ab initio MD study (see ref. 49), it has been suggested that such relationship may break down for some systems, such as for water on two-dimensional materials. Here, we provide further evidence of such decoupling between slippage and wetting by estimating separately the contact angle by means of the theory of hydrophobicity and the friction computed with linear-response theory. For example, for both graphene and MoS_2_, we obtain a contact angle around 90 degrees, in spite of the fact that the friction on MoS_2_ is about 10 times larger compared to graphene. Further, whereas Au(111) is more wetting than Au(100), water slips faster on Au(111) than in Au(100) (Figs. 4*B* and 5*B*).

By combining the substrate-specific hydrophobic adsorption and solid/liquid friction, we explore the diffusio-osmotic transport of hydrophobic particles, represented by their diffusio-osmotic mobility $D_{\mathrm{DO}}$. Fig. 5 *C* and *D* illustrates the diffusio-osmotic mobility as a function of the bulk density of a given hydrophobic solute $n_{s}^{\;\text{b}}$, as a function of its Gibbs adsorption length $K_{s}$ and the water Gibbs adsorption length $K_{w}$ under no-slip and slip conditions. Three notable effects emerge: First, a pronounced slip-induced amplification of the diffusio-osmotic mobility (59) is observed, leading to an approximately one-order-of-magnitude increase in $D_{\mathrm{DO}}$ compared to the no-slip case for high-slip substrates like graphene. In this scenario, the slip contribution in Eq. **9** dominates over the nonslip term due to the large slip length on graphene, resulting in $D_{\mathrm{DO}}∼b\left(K_{s}-K_{w}\right)$. Second, there is an approximately linear dependence between $D_{\mathrm{DO}}$ and $\left(K_{s}-K_{w}\right)$. This relationship is equivalent to a linear dependence on the hydrophobe radius R, given that the dominant contribution to $K_{s}$ primarily arises from the surface term. In the dilute limit, the spatial distribution of a hydrophobe can be approximated as that of a single hard sphere approaching a hard wall, leading to $K_{s}\approx -R$ and therefore a linear dependence of $D_{\mathrm{DO}}$ on the hydrophobe radius. Third, the sign of the diffusio-osmotic mobility, representing the direction of the diffusio-osmotic flow parallel to the surface, flips from positive to negative on graphene as the hydrophobe radius increases. This sign change implies that smaller hydrophobic particles generate a diffusio-osmotic flow from high to low solute concentration ($D_{\mathrm{DO}}>0$), whereas larger particles generate a flow from low to high solute concentration ($D_{\mathrm{DO}}<0$), as observed in diffusio-osmosis of electrolyte solutions at silica (60).

Similar trends are observed for the other substrates characterized by significant slip lengths, such as MoS_2_ and Au(111). However, neither of these substrates exhibits a change in the sign of $D_{\mathrm{DO}}$ for hydrophobic particles with R≥1.75 Å, although they get close to a turning point. The increase in $D_{\mathrm{DO}}$ is more pronounced on Au(111), due to the higher slip length compared to MoS_2_. Nonetheless, the increased friction on both Au(111) and MoS_2_, when compared to graphene, results in a nearly tenfold decrease in the scaling effect of the slip length on $D_{\mathrm{DO}}$ overall. In the case of the platinum substrates Pt(111), and Pt(100), the pronounced depletion of hydrophobes in proximity to the surface, and thus the dominant effect of $K_{s}$, results in a negative contribution to the diffusio-osmotic mobility. Due to the generally low slip lengths on these substrates, there are only significant effects on $D_{\mathrm{DO}}$ for Au(111), where the absolute value exceeds both Pt surfaces by slip amplification. Contrary to the above materials, the diffusio-osmotic flow on the Au(100) surface is constant from high to low concentration as $D_{\mathrm{DO}}>0$ with minor dependence on the hydrophobe size. This peculiar transport arises from the interplay of the low surface hydrophilicity and the apparent size-independent adsorption of hydrophobes. These results demonstrate a remarkable dependence of the diffusio-osmotic flow on the Au surface orientation.

## Conclusion

Our investigation utilizes advanced neural network potentials to disentangle intricate relationships between substrate materials, interfacial water structure, and transport properties at several technologically important water/solid interfaces through extensive molecular dynamics simulations. We reveal substrate-induced structural effects ranging from a uniform water layer on graphene to a highly structured layer on Pt(100), highlighting the diversity of solid–liquid interactions. Consequently, these substrate-dependent structural features impact the characteristic adsorption of hydrophobic solutes in the interfacial region, which contributes to macroscopic wetting behavior, as characterized by the contact angle. Furthermore, variations in the dynamic behavior of the water contact layer, which manifest as capillary wave fluctuations at the interface, are presented as an equally consistent measure of wettability. Finally, we relate properties of the substrate-dependent water contact layer which significantly contribute to water slippage to the enhancement of diffusio-osmotic mobilities. This work builds upon previous research on liquid/solid interface hydration (e.g. refs. 7, 31, and 33) and on the connection between wetting, slippage, and osmotic transport (e.g. refs. 32, 58, and 59). It connects microscopic structural and dynamical properties of the liquid/solid interface to macroscopic properties that are relevant for the development of surface coatings, nanofluidic systems, and filtration membranes.

## Materials and Methods

### Production Molecular Dynamics Simulations.

Extensive MD simulations of water-filled slits under confinement are used to investigate the structure and dynamics of liquid water in contact with graphene, MoS_2_, Au(100), Au(111), Pt(100), and Pt(111), with the liquid being about 7 nm to 8 nm thick. The water film thickness is sufficiently large to recover bulk behavior in the computed structural properties far from the surfaces. The simulation cell dimensions in the direction parallel to the surfaces vary between 2.9 nm to 3.5 nm side lengths, depending on the molecular structure of each material. The Pt and Au systems are modeled with four layers, with the last two truncated to their bulk structure, whereas single-layer graphene and single-layer MoS_2_ are used for the two-dimensional materials. Periodic boundary conditions are applied in all dimensions and the interface systems are separated by a vacuum region of 40 Å in the direction perpendicular to the surface. The results presented in this work are obtained from MD simulations performed with the Large-scale Atomic/Molecular Massively Parallel Simulator (LAMMPS) code (61) that were run in the canonical ensemble at 330 K for 5 ns using C-NNPs. Further details on the MD simulations are discussed in *SI Appendix*.

### Training of the HDNNPs and Ab Initio Simulations.

The MD simulations are performed using Committee Neural Network Potentials trained on extensive ab initio simulations (18), where each committee consists of eight independent Second Generation HDNNP (62). The HDNNPs are based on atomic-centered symmetry functions and have been demonstrated to accurately describe the phase diagram and the structure of water (63, 64), complex solid/water interfaces (65) and reactive processes (66). Four individual C-NNPs have been trained, one for each type of liquid/solid interface, and they result from an iterative process based on active learning, where the training set size increases and the structural diversity improves at each subsequent iteration. The final models are obtained after 5 to 6 iterations. A brief introduction into C-NNPs and HDNNPs followed by a detailed account of the training and validation process, including a comparison on the structural properties computed from explicit ab initio MD simulations, is given in *SI Appendix*.

All ab initio simulations used to train the C-NNPs have been carried out with CP2K (67), using the Gaussian and plane wave method (68). Further, the ab initio MD trajectories used in the initial iteration of the training process are taken from ref. 50 for the Water/MoS_2_ and Water/Graphene interfaces and from ref. 69 for bulk water, and were obtained with the optB88-vdW functional (70, 71). Instead, the initial ab initio MD trajectories for the Water/Au and Water/Pt systems were performed by following ref. 25 and were obtained using 2nd generation Car–Parrinello MD simulations (72) employing the PBE+D3 (73, 74) functional. The final training set for the C-NNPs consists of ab initio of total energies and forces computed from single-point calculations, which were performed for all systems using the optB88-vdW functional with DZVP-MOLOPT-SR-GTH basis sets and with a cutoff between 1,000 and 1,200 Ry for the expansion into plane-waves (75).

Despite the challenges of modeling water and aqueous interfaces with DFT (15, 76), the optB88-vdW (70, 71) functional has demonstrated to describe the structure of bulk water in agreement with experiments (77) and with accurate Post–Hartree–Fock methods such as MP2 (78). Dynamical properties, and specifically, the diffusion coefficient and the viscosity are also accurately described at temperatures above 300 K and above (69). Additionally, the optB88-vdW functional predicts interaction energies of water on graphene within 50 meV of that predicted with Diffusion Monte Carlo (79) and can capture the stability of low dimensional water structures on noble metal surfaces relative to ice-Ih (80). Further, the structure and dynamics of liquid water on solid surfaces is in close agreement with other dispersion-corrected functionals that have also shown to describe aqueous interfaces accurately. For example, the water density profiles on Au(111) and on Pt(111) predicted using our C-NNPs trained on optBBB-vdW agree well with that obtained from ab initio MD trajectories obtained with the PBE+D3 functional (25) (*SI Appendix*). Analogously, the friction coefficient of water on graphene predicted with optB88-vdW (49) is in good agreement with MD simulations using machine learning force fields trained using the revPBE+D3 functional (53). All input files, model files for the different C-NNPs and the datasets are available at ref. 81.

### Structuring and Positional Order in the Contact Layer.

The density profile is a fundamental property to quantify the structuring of water in the direction perpendicular to the surface and it is defined as

[1] $$\begin{array}{c} \rho \left(z\right)=\frac{1}{V}\langle \sum_{i}m_{i}\delta \left(z-z_{i}\left(t\right)\right)\rangle , \end{array}$$

where $z_{i}\left(t\right)$ is the instantaneous height of an atom in the liquid with mass $m_{i}$ from the nearest surface atom and V the volume of the thin layer over which is averaged. The water density profile is also instrumental to define the contact layer, that here is defined as the region up to the first global minimum in ρ(z), which is reached at a height from the surface between 0.4 nm and 0.5 nm for the systems under investigation (see the colored peaks in Fig. 1). Additionally, the region between the global density minimum and the following minimum away from the surface is referred to as the transition layer.

A 2D-PCF is obtained by sampling the 2D distance vector $\Delta r_{\mathrm{ij}}=r_{i}-r_{j}$, where only the component parallel to the surface is considered, Δr=(Δx,Δy). The distribution function for the oxygen belonging to the contact layer becomes (82)

[2] $$\begin{array}{c} g_{\;\text{OO}}\left(\Delta r\right)=\frac{1}{N\rho }\langle \sum_{i\neq j}\delta \left(\Delta r-\Delta r_{\mathrm{ij}}\left(t\right)\right)\rangle , \end{array}$$

where N and ρ are the average number and the average surface density of water molecules in the contact layer, respectively. Also instrumental to characterize ordering and the corrugation of the energy landscape in the contact layer, is the two-dimensional FES, which is defined as

[3] $$\begin{array}{c} \Delta G\left(x,y\right)=-k_{B}T\log\left[P\left(x,y\right)/P_{\;\text{max}}\right], \end{array}$$

where P(x,y) is the probability to find a water molecule in the contact layer at lateral coordinates (x,y) and $P_{\;\text{max}}$ is its maximum value. An important connection between the 2D-PCF and the FES is established in the long-distance limit to analyze the long-range order (83). In the limit Δr→∞, the 2D-PCF tends to the single particle density distribution, which is proportional to $\exp\left[-\Delta G_{\mathrm{Oxy}}\left(x,y\right)/k_{B}T\right]$, where $\Delta G_{\mathrm{Oxy}}\left(x,y\right)$ is the 2D FES experienced by the oxygen atoms in the water contact layer. It can be seen that, for a two-dimensional liquid with short-range order, the ideal-gas limit of the 2D-PCF is recovered, i.e. $g_{\;\text{OO}}\left(\Delta r\to \infty \right)\to 1$. This means that the 2D-PCF of the water contact layer at the interface with a crystalline surface does not tend to the ideal gas limit, because the surface acts as an external potential, effectively inducing long-range order in the fluid.

### Hydrophobic Hydration at Solid/Liquid Interfaces.

Macroscopically, the hydration of a solid surface is described by the contact angle of a water droplet, which then characterizes its hydrophilicity/hydrophilicity. Instead, microscopic fingerprint of hydration is provided by the solvation energy of a cavity, as described in a number of pioneering works (7, 8, 29, 84,86–87). Hydrophobic hydration is in fact a multifaceted effect involving multiple length-scales that become relevant depending on whether one considers the hydration of small molecules, of clusters of molecules, of extended solid surfaces or of soft interfaces (30).

In the case of a liquid water interface adjacent to an extended surface, hydration can be characterized by analyzing the magnitude of the capillary wave fluctuations of the instantaneous liquid surface. The structure of water on a hydrophobic substrate resembles that of the liquid–vapor interface, such that the weaker the interactions between water and the substrate the larger the capillary wave fluctuations (87). Thus, we characterize the hydration of the contact layer through the examination of the height distribution of the instantaneous dividing interface, which delineates the separation between the solid and the liquid phases. The calculation of the instantaneous dividing interface is performed using the Willard–Chandler method (88).

To characterize the hydration of the different substrates, the height distribution of the instantaneous dividing interface is computed as

[4] $$\begin{array}{c} P\left(\Delta z\right)=\langle \delta (h(t)-\Delta z)\rangle , \end{array}$$

where h(t) is the height of the instantaneous dividing interface with respect to the underlying substrate and $\Delta z=z-\bar{h}$ is the displacement along the surface normal from the average height of the instantaneous dividing interface $\bar{h}$. Specifically, h(t) is expressed by the height difference between the instantaneous liquid surface $h_{\mathrm{liquid}}\left(t\right)$ and the instantaneous solid surface $h_{\mathrm{solid}}\left(t\right)$ at each grid point. The snapshot in Fig. 4*D* shows the liquid (blue) and solid (orange) instantaneous surfaces for the graphene solid/liquid interface as an example.

To characterize the adsorption of small hydrophobic solutes at the aqueous interface, such as CO, N_2_, CH_4_, or H_2_, we calculate the free energy associated to the formation of a cavity at the interface using a spherical probe volume (8, 30, 31, 85). We define the free energy of solvation of a hydrophobe of radius R at the height z from the surface as

[5] $$\begin{array}{c} \delta \mu \left(z\right)=-k_{B}T\log\left[P\left(z,N_{R}=0\right)/P\left(z^{\;\text{b}},N_{R}=0\right)\right], \end{array}$$

where $P(z,N_{R}=0)$ is the probability to find an empty spherical volume af radius R at z, $z^{\;\text{b}}$ is the height at which the free energy reaches its bulk value and $k_{B}T$ is the thermal energy. Throughout this work, the free energy of adsorption of the hydrophobes is computed using Eq. **5**, where the bulk free energy is reached at about 2.0 nm from the surface. The thin vacuum-like region of about 0.2 nm to 0.3 nm that forms between the surface and the water contact layer may lead to the stabilization of cavities in this region. The surface is considered a hard wall with respect to hydrophobic adsorption, following the condition δμ(z<R)→∞.

The characteristic adsorption of hydrophobes of different diameters at the respective interfaces can directly be related to the substrate-specific water contact angle (33). To obtain the water contact angle θ, the minimum free energy of adsorption at the interface $\delta \mu (z^{*})$ is related to the corresponding bulk free energy $\mu_{b}$ by

[6] $$\begin{array}{c} \delta \mu \left(z^{*}\right)=m\left[\cos\left(\theta \right)-\cos\left(\theta_{0}\right)\right], \end{array}$$

with $m=\mu_{b}/2\left(1+\cos\left(\theta_{0}\right)\right)$ and $\theta_{0}=45^{{}^{\circ}}$ as the boundary between hydrophobic and hydrophilic interactions.

### Nanofluidic Slip.

We consider the relative motion of water in contact with crystalline surfaces assuming the fluid is incompressible with a bulk viscosity η. Slip in the direction parallel to the surface is described by the partial slip boundary condition for the velocity profile of the fluid as a function of the height from the surface v(z), $b\partial_{z}v\left(z\right){{|}_{z=0}=v\left(z\right)|}_{z=0}$, with b the slip length which is defined as the distance at which the extrapolation of the velocity profile in the solid becomes zero (55). The slip length is inversely proportional to the solid/liquid friction coefficient λ according to b=η/λ. λ is extracted from equilibrium MD simulations by computing the friction coefficient under linear response theory according to the following Green–Kubo expression (89):

[7] $$\begin{array}{c} \Lambda \left(t\right)=\frac{1}{2Sk_{B}T}\int_{0}^{t}\langle F\left(0\right)\cdot F\left(t^{\prime }\right)\rangle dt^{\prime }\;, \end{array}$$

with S the surface area of the solid in contact with the fluid and F(t) the instantaneous force exerted on the solid in the direction parallel to the surface. F(t) is obtained as the sum of the forces on the individual atoms in the liquid. The equilibrium value of the friction coefficient should be obtained as the value of the running integral in Eq. **7** when it reaches a plateau. However, the value of the Green–Kubo expression vanishes in the long-time limit as a consequence of the finiteness of the water slab. Thus, we compute the equilibrium value of the friction coefficient by fitting the following analytical expression derived in Oga et al. (90) for finite-size systems,

[8] $$\begin{array}{c} \Lambda \left(t\right)=\lambda_{0}\left(e^{-\frac{t}{t_{1}}}-e^{-\frac{t}{t_{2}}}\right), \end{array}$$

where $\lambda_{0}$, $t_{1}$, and $t_{2}$ are fitting parameters. The friction coefficient can then be obtained from $\lambda =\lambda_{0}\left(1-u\right)/\left(1+u\right)$, with $u=t_{2}/t_{1}$.

### Diffusio-Osmosis of Hydrophobic Solutes.

Assuming linear response theory, the diffusio-osmotic velocity of the fluid in the bulk of the channel $v_{\mathrm{DO}}$ is proportional to the applied solute concentration gradient according to the relation $v_{\mathrm{DO}}=-D_{\mathrm{DO}}\left(-\nabla n_{s}^{b}/n_{s}^{b}\right)$, where $D_{\mathrm{DO}}$ is the diffusio-osmotic mobility (59). A relation for $D_{\mathrm{DO}}$ can be obtained starting from the Stokes equation $-\eta \partial_{z}^{2}v\left(z\right)=f\left(z\right)$, where f(z) is the force density arising from the applied concentration gradient. The force density can in turn be expressed in terms of the bulk-normalized number density of the solutes (i.e. $n_{\;\text{s}}\left(z\right)/n_{\;\text{s}}^{\;\text{b}}$), and of the water (i.e. $n_{\;\text{w}}\left(z\right)/n_{\;\text{w}}^{\;\text{b}}$) according to the Gibbs–Duhem relation (91, 92): $f\left(z\right)=\left[n_{s}\left(z\right)-n_{s}^{b}n_{w}\left(z\right)/n_{w}^{b}\right]\left(-k_{B}T\nabla n_{s}^{b}/n_{s}^{b}\right)$. Integrating the Stokes equation with the partial slip boundary condition and introducing the characteristic length-scales for water, $K_{w}$ and $L_{w}$, and for the solute, $K_{s}$ and $L_{s}$, leads to the following expression for the diffusio-osmotic mobility $D_{\mathrm{DO}}$:

[9] $$\begin{array}{c} D_{\;\text{DO}}=-\frac{v_{\mathrm{DO}}}{-\nabla n_{s}^{b}/n_{s}^{b}}=\frac{k_{B}T\times n_{s}^{\;\text{b}}}{\eta }\left[K_{s}L_{s}-K_{w}L_{w}+b\left(K_{s}-K_{w}\right)\right], \end{array}$$

where $K_{w}=\int_{0}^{\infty }\left[n_{w}\left(z\right)/n_{w}^{\;\text{b}}-1\right]dz$ and $K_{s}=\int_{0}^{\infty }\left[n_{\;\text{s}}\left(z\right)/n_{\;\text{s}}^{\;\text{b}}-1\right]dz$ are the Gibbs adsorption lengths of the water and of the hydrophobic solutes, respectively. The larger (smaller) the Gibbs adsorption length the more attracted (repelled) the liquid or the solutes are to the surface, where we note that $K_{w}$ and $K_{s}$ can both be either positive or negative. Additionally, the length-scales $L_{w}=K_{w}^{-1}\int_{0}^{\infty }z\left[n_{w}\left(z\right)/n_{w}^{\;\text{b}}-1\right]dz$ and $L_{s}=K_{s}^{-1}\int_{0}^{\infty }z\left[n_{\;\text{s}}\left(z\right)/n_{\;\text{s}}^{\;\text{b}}-1\right]dz$ define the mean width of the interface where nanoscale density oscillations are relevant, for water and for the hydrophobic solutes, respectively (93).

Analogous equations have been recently derived for diffusio-osmosis due to a salt concentration gradient (92), while here the focus is on diffusio-osmotic transport due to the concentration gradient of a neutral solute. In particular, we focus on diffusio-osmotic flow due to concentration gradients of ideal hydrophobic solutes in the dilute regime (30). Therefore, $D_{\;\text{DO}}$ can be computed directly from equilibrium MD simulations, where besides the slip length (*Hydrophobic Hydration at Solid/Liquid Interfaces*), the central quantities required to calculate $D_{\;\text{DO}}$ are the normalized water density profile $n_{\;\text{w}}\left(z\right)/n_{\;\text{w}}^{\;\text{b}}$ and the solute density profile which is explicitly obtained from Eq. **5**, namely $n_{\;\text{s}}\left(z\right)/n_{\;\text{s}}^{\;\text{b}}=\exp\left[-\delta \mu \left(z\right)/k_{B}T\right]$.

We conclude this section with a brief discussion of the assumptions underlying our description of hydrodynamics and of diffusio-osmosis. In this work, we focus on slipping (e.g. graphene) as well as nonslipping interfaces, where the water contact layer is tightly bound to the surface [e.g. Pt(111) and Pt(100)]. We remark that the partial slip boundary condition is valid in both cases, since in the limit of b→0 the no-slip boundary condition of continuum hydrodynamics is recovered. Further, we assume that the viscosity of water η is constant and equal to its value in the bulk fluid. Although this assumption holds for the description of electrokinetic effects at hydrophobic surfaces, on hydrophilic surfaces the increase in viscosity relative to bulk conditions can be incorporated into the description of slippage in terms of an effective slip length (94). This correction leads to a reduction of the effective slip length itself on hydrophilic surfaces. Here, we consider two limiting cases to account for the possibility that the diffusio-osmotic mobility on hydrophilic surfaces, such as Pt(111) and Pt(100), may be overestimated: one that assumes that the slip length is zero (i.e. no-slip boundary condition) and the $D_{\mathrm{DO}}$ is only given by the first two terms in Eq. **9**, and one where it is instead calculated from b=η/λ, where η is taken to be the experimental viscosity of bulk water (i.e. partial-slip boundary condition).

## Supplementary Material

Appendix 01 (PDF)

## Data Availability

All input files, model files for the different C-NNPs and the datasets have been deposited in Zenodo (https://doi.org/10.5281/zenodo.12774464) (81). Previously published data were used for this work (25, 50, 69).
