# Supplementary material for: The role of the water contact layer on hydration and transport at solid/liquid interfaces
Source: Proc Natl Acad Sci U S A. 2024 Sep 11;121(38):e2407877121. doi: 10.1073/pnas.2407877121 (PMC11420213; doi:10.1073/pnas.2407877121)
Supplement: Supplementary file 1 — Appendix 01 (PDF) [file pnas.2407877121.sapp.pdf]

Supporting Information for

The role of the water contact layer on hydration and transport at solid/liquid interfaces

J Gäding, V. Della Balda, J. Lan, J. Konrad, M. Iannuzzi, R. Meißner and G. Tocci

Corresponding Authors M. Iannuzzi, R. Meißner
E-mail: marcella.iannuzzi@uzh.ch
E-mail: robert.meissner@tuhh.de

This PDF file includes:

Supporting text
Figs. S1 to S15
Tables S1 to S2
SI References

Supporting Information Text

1. Workflow for the committee neural network potentials

This section provides a detailed description of how the Committee Neural Network Potentials (C-NNPs) for aqueous interfaces were developed. The C-NNPs were designed to describe the atomic-scale interactions at the level of density functional theory (DFT). The focus is on water at the interface with 2D materials (graphene and MoS_2) and on water at the interface with noble metals [Pt(100), Pt(111), Au(100) and Au(111)]. These specific interfaces were chosen as they represent model systems in the fields of electrochemistry and nanofluidics and, among other technologies, they are relevant to solar-to-fuel energy conversion, as well as for osmotic-energy conversion.

In general, a C-NNP consists of an ensemble of multiple neural network potentials, where the training and validation sets of each neural network are obtained by randomly selecting 90% of the underlying dataset for training and 10% for validation. This random selection process is applied individually for each neural network potential. Following the approach of Ref. (1), each C-NNP in this work consists of 8 neural network potentials. The workflow to build a C-NNP is inspired by the approach introduced by Refs. (1, 2) and makes use of the Active Machine Learning (AML) package (see https://github.com/MarsalekGroup/aml) and of the `n2p2`-library (3). The complete workflow is illustrated in Fig. S1 and is discussed below. Additionally, all input files, model files for the different C-NNPs and the datasets are available at Ref. (4):

Step 1 The first step is to generate a dataset of atomic configurations, total energies and forces from *ab initio* molecular dynamics (AIMD) simulations for a given system of interest, consisting of a liquid/solid interface and of bulk water. In the present work, AIMD data generated in previously published work is utilized and subsequently refined.

Step 2 Secondly, if the solid under investigation is a metal, a single NNP is trained and NNP-MD is carried out to explore a wider configuration space than covered by the first AIMD trajectory. Subsequently, the workflow proceeds with an active learning step, specifically using the Query by Committee (QbC) method (2). For 2D materials and for bulk water, the QbC step follows immediately after the sampling with AIMD.

The QbC approach is employed on each trajectory individually to identify a set of diverse structures that can be used to build a first stable (albeit not necessarily accurate) interatomic potential that is suitable to explore a wider configuration space. During the initial QbC step, 40 randomly selected configurations are sampled from the provided trajectory. These randomly sampled configurations are used as a reference set of a preliminary C-NNP composed of 8 neural networks within the QbC cycle. Subsequently additional 20 structures are added to the training set based on the largest mean force disagreement within the preliminary C-NNP model. This process is repeated iteratively until approximately 140 structures are selected or no further relevant structures are identified in the given trajectory. Because preliminary C-NNPs are only used to select the most suitable structures and therefore do not need to have the same accuracy as the final C-NNP, they are trained over only seven epochs. By combining all the QbC-sampled configurations from the provided trajectories, the first initial set for the training of the C-NNP model is obtained. Further details on the composition and size of each dataset, as well as the type of *ab initio* calculations performed, are found in the section pertaining to each system under investigation. See Sec. A – D. Additionally, refer to *Training of the HDNNPs and Ab Initio Simulations* in the *Materials and Methods* section of the main text for more information on the *ab initio* calculations.

Step 3 After QbC, the quality of the generated dataset is improved by performing single point DFT calculations of total energy and forces using tight DFT settings, especially concerning the use of a large cutoff of 1200 Ry in the case of 2D material interfaces, respectively 1000 Ry in the case of metal interfaces, for the auxiliary expansion of the basis-sets to plane waves. The initial set of configurations, selected via QbC, along with corresponding forces and energies from the *ab initio* single point calculations serves as the reference set for the training of the initial C-NNP model.

Step 4 A first-generation C-NNP model is obtained by training of the committee of neural networks for 15 epochs to guarantee adequate convergence, according to recent studies (1, 5). Upon completion of training, molecular dynamics simulations are carried out for each system using the current C-NNP. During the C-NNP MD runs, positions, forces, and committee disagreement are sampled every 100 fs, as well as every configuration that leads to extrapolations in any symmetry function. The MD runs continue until a simulation time of 500 ps is reached or 50000 configurations causing extrapolations are obtained. In cases where the system becomes unstable due to nonphysical behavior, such as reconstructions or degradation, the run is terminated, and only configurations with reasonable structure are considered for further processing.

Step 5 To validate the models a number of properties are compared with *ab initio* results. Some properties vary depending on the respective material and include the coefficient of friction for 2D materials, or general values such as the RMSE of per-atom forces for example. Section E summarizes the validation of the C-NNPs models. If criteria are not satisfied, it is necessary to extend the reference set with additional structures. First, 60 non-continuous configurations are selected based on their G-function values according to equation 1.

$$\Delta G = \sum_{G_i < G_i^{\min} \vee G_i > G_i^{\max}} \left| \frac{G_i - G_i^{\min}}{G_i^{\max} - G_i^{\min}} - \frac{1}{2} \right| - \frac{1}{2} \qquad [1]$$

with G_i as the actual value of any of the ith ACSFs at a give configuration, $G_i^{\max}$ and $G_i^{\min}$ as the upper and lower limit of the specific ACSF G_i, respectively. The metric takes into account the deviation of the symmetry functions from their limits and the number of atoms affected, according to the extrapolation metric proposed by Ref. (6).

Subsequently, if fewer than 60 configurations can be sampled using the extrapolation metric ΔG, the remaining number of structures are selected based on their committee disagreement. The disagreement is given by

$$\sigma E_{\mathrm{C-NNP}} = \sqrt{\frac{\sum_1^{N_{\mathrm{NNP}}}(E_{\mathrm{NNP},i} - \bar{E}_{\mathrm{NNP}})^2}{N_{\mathrm{NNP}}}}, \qquad [2]$$

where $E_{\mathrm{NNP},i}$ is the total energy of the ith committee member NNP, $\overline{E}_{\mathrm{NNP}}$ is its average over all committee members, and N_{NNP} is the total number of committee member NNPs. Prior to their inclusion in the training set, *ab initio* single point calculations are carried out for each of the sampled configuration. The process of training, continuing molecular dynamics runs using the updated C-NNP model and subsequental validation is repeated until the C-NNP model reaches the desired level of accuracy.

A. C-NNP Training: Graphene. A comprehensive summary of the composition of each generation of the C-NNPs for the Water/Graphene interface is illustrated in the top Figure S2. An initial dataset was generated using QbC starting from AIMD trajectories from Ref. (7) which were deposited on Ref. (8). These AIMD trajectories consist of water confined between two graphene sheets with varying fluid thicknesses, between 0.9 nm and 2.2 nm. A 1st generation C-NNP was trained on a total of 435 configurations which were selected via the QbC approach. These consist of confined water systems with fluid thicknesses of 0.9 nm, 1.5 nm, and 2.2 nm each containing 138, 143 and 154 structures, respectively, and with graphene sheets of size (5×3) expressed in terms of a rectangular unit cell which is replicated 5 times in the direction parallel to the zig-zag direction and 3 times in the direction parallel to the arm-chair direction. After training, the 1st generation C-NNP was used to perform MD simulations at 330 K on the following systems: bulk water containing 64 water molecules, water confined between two graphene sheets with fluid thicknesses between 0.9 nm and 2.5 nm and on (5×3) cells, as well as on larger (10×6) cells. From each trajectory, 10 structures with the highest committee disagreement were selected to extend the dataset further, and a total of 495 structures were used to train the 2nd generation model. MD simulations with this model were then performed at three different temperatures, specifically at 260 K, 330 K and 400 K, and 20 configurations for each temperature and for each system were selected according to the committee disagreement. However, configurations for confined systems with (5×3) cells were removed from the dataset, to avoid potential finite size effects that may affect the training of the next generation C-NNP. The training of the 3rd generation C-NNP was then performed on a total of 480 structures, which contained larger (10×6) Water/Graphene and bulk water configurations sampled at different temperatures. For the 4th generation model, 500 K was considered as an additional simulation temperature and 640 structures with highest disagreement were added to the dataset for a total of 1120 configurations. The final 5th generation model was achieved by adding 20 extrapolation structures taken from trajectories of all interface systems at 330 K. The final reference set of the C-NNP graphene model consists of 1260 structures.

B. C-NNP Training: MoS_2. The different generations of the C-NNPs for the Water/MoS_2 interface are illustrated in the bottom Figure S2. The dataset for this system is also based on AIMD trajectories from Ref. (7), which are available at Ref. (9). The first C-NNP model was trained on 142 configurations of water confined between two MoS_2 sheets and of 133 configurations of a water film on a MoS_2 sheet selected via the QbC approach. MD simulations were then performed with the 1st generation C-NNP model to further explore the configuration space of both systems. Using the extrapolation metric and committee disagreement in Eqs. Eq. (1) and Eq. (2), 20 snapshots were sampled from each trajectory and added to the dataset. Additionally, an MD simulation of bulk water box with 64 molecules was carried out with the same model to yield another 20 extrapolation snapshots. The 2nd generation C-NNP was thus obtained by performing single-point DFT calculations on a total of 335 configurations sampled with MD using the 1st generation C-NNP. The two subsequent model generations (*i.e.* the 3rd and 4th generation) were obtained in the same way, by adding 40 configurations for each system, for a total of 120 configurations for each generation. For the 5th and 6th generation C-NNPs, instead 60 and 180 configurations for each system were introduced in the dataset, respectively. The 6th generation C-NNP model used in the production MD simulations is trained on a total of 1295 configurations.

C. C-NNP Training: Au. The top of Figure S3 illustrates the evolution of the C-NNP models from the 1st to the 6th generation for the Water/Au interface. The initial configurations for the active-learning workflow for the Water/Au C-NNP model consist of MD trajectories obtained with a pre-trained NNP model resulting from 2nd generation Car-Parrinello MD (10) simulations with the PBE-D3 functional (for more details on the computational settings of the 2nd generation Car-Parrinello MD for the Water/Metal interface systems see Ref. (11)). An additional bulk water AIMD trajectory with the optB88-vdW functional from Ref. (12) containing 32 water molecules was included to the initial configurations. Five different types of systems were sampled with MD to generate each C-NNP model: a bulk water system, clean Au(111) and Au(100) slabs, and water-covered Au(111) and Au(100) surfaces. For the (100) and (111) surfaces, 8×8 supercells were used and for the aqueous Au interfaces film thicknesses of 3.5 nm [in case of Au(111)] and 3.2 nm [in case of Au(100)] were used. The training of the 1st generation C-NNP model was carried out on 334 configurations selected with the QbC approach, and where the total energies and forces

were recomputed with tight DFT settings using the optB88-vdW functional. To obtain the 2nd generation C-NNP model, configurations were selected via the committee disagreement and extrapolation metric from MD trajectories performed at 330 K using the C-NNP from the 1st generation C-NNP model. Single-point DFT calculations of total energies and forces were then performed prior to training the next generation model. From the 2nd to the 6th generation, the bulk water MD simulations were carried out with 256 molecules and no additional clean surface simulations were performed. This process was repeated until the 6th generation, to obtain a dataset of 1234 structures.

D. C-NNP Training: Pt. The bottom of Figure S3 illustrates the evolution of the C-NNP models from the 1st to the 6th generation for the Water/Pt interface. The active-learning workflow follows similar steps as with the Water/Au interface. The initial configurations are obtained from MD trajectories with a NNP model that is pre-trained on 2nd generation Car-Parrinello MD trajectories (see Ref. (11) for more details). An additional bulk water AIMD trajectory with the optB88-vdW functional from Ref. (12) containing 32 water molecules was included to the initial configurations. MD simulations were then run for five different systems to generate each C-NNP model: a bulk water system, clean Pt(111) and Pt(100) slabs, and water-covered Pt(111) and Pt(100) surfaces. For the (100) and (111) surfaces, 8×8 supercells were used and a film thickness of 2.3 nm was used for the water-covered Pt interfaces. The training of the 1st generation C-NNP model was carried out on 393 configurations selected with the QbC approach, and where the total energies and forces were recomputed with tight DFT settings using the optB88-vdW functional. The configurations used to train the 2nd generation C-NNP model were selected by sampling each system with MD simulations using the previous generation C-NNP model at three different temperatures: 260 K, 400 K, and 330 K. In this step, the number of molecules used in the bulk water simulations was increased to 256, while no additional simulations were performed on clean surface systems. Configurations were then selected via the committee disagreement and extrapolation metric, and single-point DFT calculations of total energies and forces were performed prior to training the next generation model. This process was repeated until the 6th generation, to obtain a dataset of 1233 structures. However, we note that from the 3rd to the 6th model, the MDs were carried out at 330 K. Finally, the 6th generation C-NNP model for the aqueous Pt interface was trained on 1233 structures.

E. C-NNP model validation . To validate the accuracy of the individual C-NNP models, the results obtained for 60 sampled structures (with the extrapolation and σ-criterion as described in Sec. 1) are compared with *ab initio* results. First the root mean squared error (RMSE) of the per-atom forces is calculated. As a threshold a RMSE(F_i) of 75.0 meV/Å is set according to previous studies employing NNPs (1, 2, 5). The C-NNP models meet said accuracy criteria in their last generation which is then used for the production MD runs, as can be seen in the the values of the RMSEs summarized in Figure S4(a).

The RMSE of the potential energy per atom, given by $E_{\text{pot}}/n_{\text{atoms}}$, is used as a second validation criterion. The threshold for each C-NNP model was set to RMSE($E_{\text{pot}}/n_{\text{atoms}}$) $\leq$ 2.0 meV, according to Refs. (1, 2, 5). Figure S4b summarizes the RMSE($E_{\text{pot}}/n_{\text{atoms}}$) values of each C-NNP model in reference to its *ab initio* calculations. Overall, each model is below the desired threshold and has a comparable accuracy to a recent work of Ref. (1). Although not employed as a critical validation criteria in this work, the ratio between the number of configurations leading to extrapolations relative to overall trajectory frames is given in Figure S4c. Remarkably, this ratio has proven to be a useful parameter to measure the stability of a C-NNP model, especially in the early stages of training. In the course of the training, however, this value should steadily decrease. Since the value varies depending on the size of the system, i.e., larger systems with more atoms have a higher chance of leading to extrapolations than smaller systems, it is not directly used as a criterion to terminate the training loop shown in Fig. S1, but it provides a useful guideline to gauge the overall robustness and stability of the model.

After the validation step through the analysis of the RMSE(F_i) and RMSE($E_{\text{pot}}/n_{\text{atoms}}$), specific structural and dynamical properties are compared with *ab initio* results. First, we compare the oxygen-oxygen radial distribution function $g_{\text{OO}}(r)$ of bulk water extracted from separate C-NNP MD trajectories, one for each model, with the reference AIMD trajectory of Ref. (12) obtained with the optB88-vdW functional (see Figure S5). The system size, temperature, timestep and overall length of the C-NNP trajectories are identical to the AIMD trajectory. Specifically, in the figure the $g_{\text{OO}}(r)$ from AIMD simulations with 32 water molecules in Ref. (12) are compared with the RDFs from C-NNP MD simulations with the same system size, as well as with larger boxes containing 256 water molecules. The reason for this is, on the one hand, to compare the AIMD data and the C-NNP MD results on equal footing using the same number of water molecules, and on the other hand, to show that the model can generalize well to large bulk water simulations. It can be seen that the radial distribution functions predicted by each C-NNP model using 32 water molecules boxes is overall in good agreement with the radial distribution functions from AIMD trajectories with the same number of water molecules. However, some differences are visible when comparing the RDFs with boxes containing 32 water molecules between the different C-NNP models due to the small box size, in particular in the height of the first peak. Nevertheless, these differences disappear in the RDFs obtained from C-NNP MDs computed on the larger boxes containing 256 water molecules. Thus, whereas the RDFs with the C-NNP MDs on smaller boxes present some finite-size effects and exhibit some differences between the different models, the RDFs on larger boxes are free from such finite-size effects.

Second, for each C-NNP model, we compute the diffusion coefficient D of bulk water and also compare it with the reference AIMD data in Ref. (12), in order to verify the accuracy of the description of water dynamics. For each C-NNP model a separate bulk water trajectory of 500 ps with 256 molecules are simulated. The diffusion coefficient D_{PBC} was determined from the slope of the Mean Square Displacement MSD in the diffusive regime. The transition to the diffusive regime is estimated by the intersection of linear fits in the log-log-plot of the Mean Square Displacement at around 4 ps, as illustrated in Figure S6.

To take hydrodynamic interactions between periodic image boxes into account the diffusion coefficient has to be corrected according to Equation 3 (12, 13):

$$D_{R_\mathrm{h}} = \frac{D_\mathrm{PBC}}{1 - \frac{2.837 R_\mathrm{h}}{L_\mathrm{box}}}, \quad [3]$$

where D_PBC is the diffusion coefficient computed from the MD trajectories in periodic boundary condition, L_box is the simulation box length, D_{R_h} is the corrected diffusion coefficient and $R_\mathrm{h} \sim 1.0$ Å is the hydrodynamic radius for the considered temperature (see Ref. (12)).

As a further validation step, the water density profiles for the aqueous interface systems under investigation computed with the C-NNP models are in good agreement with the reference *ab initio* MD trajectories, as seen by comparing the density profiles in Fig 1 in the main text with Fig. S8. The same is true for the two-dimensional oxygen-oxygen pair correlations obtained directly from AIMD simulations and shown in Fig. S9, that agree well with those obtained in with the C-NNP MD simulations, shown in Fig. 1 in the main text.

2. Computational details

A. High Dimensional Neural Network Potentials. In this section we give a brief introduction into Second Generation High-Dimensional Neural Network Potentials (2G-HDNNPs) and their use in Committee Neural Networks to ultimately reduce the generalization error. For further details see Refs. (14) and (2).

In contrast to initial machine learning potentials, which described the total potential energy surface (PES) of a system by means of a single neural network, HDNNPs approximate the energy contribution of each individual atom (E_i) to the total PES (E) of the system, following equation:

$$E = \sum_{i=1}^{N_\mathrm{elem}} \sum_{j=1}^{N_\mathrm{atom}^i} E_j^i \quad [4]$$

with N_elem as the number of elements considered and N_atom^i as the number of atoms of the specific element. By this means, the HDNNPs employ a independent neural network for each atom in the system, which allows its transferability between systems of varying number of atoms. In its second generation the HDNNPs are based on the locality approach proposed by Ref. (15) in 2007, by which the majority of the atomic interactions are of short ranged nature, regardless of their physical origin. Based on this assumption, the environments of an atom up to a cutoff r_c are used to describe its atomic neighborhood.

We use atom-centered symmetry functions (ACSF) to describe the atomic environments as they are rotational, translational and permutation invariant. The proposed ACSFs include two- and three-body terms in order to take radial and angular dependencies up to the cutoff radius into account. Both radial and angular ACSFs are constructed from shifted Gaussian functions combined with a monotonously decaying part of the cosine function to ensure a smooth decay to zero in both value and slope, thus allowing differentiability. In the committee approach, multiple independent optimized 2G-HDNNPs are used simultaneously as members for a single C-NNP. Although, all members of a single C-NNP share the same reference set, they diverse due to the randomized initial configuration of the weights and the stochastic nature of the optimizer. The differences between the members resulting from these small differences in the optimization can be used to estimate general errors of the C-NNP on the one hand, and to detect configurations that are insufficiently described by the C-NNP on the other hand. These configurations can subsequently be used to improve the C-NNP in a iterative training scheme as described in Sec. 1. Furthermore by averaging over each committee member a improved accuracy can be archived compared to a single 2G-HDNNP.

With respect to the number of members used per C-NNP and the underlying construction of the 2G-HDNNPs, the established values from Refs. (1) and (5) are adopted, respectively. Thus, each C-NNP consists of eight 2G-HDNNPs with a overall $r_\mathrm{c} = 6.5$ Å cutoff. The number of used ACSF varies depending on the number of elements considered in the particular system. In general, each model employs ten radial- and four angular-ACSFs per element – element relation. As example, for the Pt C-NNP (contains: Pt, O, H), this results in a total number of 54 symmetry functions per element and 162 symmetry functions in total for each 2G-HDNNP.

B. Molecular dynamics simulations. The molecular dynamics simulations (MD) utilized during the active-learning workflow to train the C-NNPs are carried out with CP2K. The cell dimensions of the systems used for training are summarized in Figs. S2 and S3. Each run consists of thus 500 ps with a timestep of 0.5 fs. The systems are kept at constant temperature using a Nose-Hoover thermostat with a relaxation time of 200 fs. Positions of the atoms, as well as the C-NNP energy and its standard deviation (σ) are sampled every 100 steps. Additionally, every configurations that cause an extrapolation in any symmetry function is saved.

The final production runs and the equilibration runs of the larger systems of slit pore confinements were conducted in LAMMPS (16). The thermostat and timestep settings used in the molecular dynamics simulations during the training phase were maintained to ensure consistency. Using slit pore confinement geometry in the final production runs allows the sampling of two solid–liquid interfaces per trajectory and thus increases the obtained statistics. Confinement heights of approx. 7.0 nm are used in order to ensure a sufficiently large bulk area in the central region of the confinements to avoid mutual interference of the solid-liquid interfaces. The surface area corresponds to 12×12 surface unit cells for each material and surface orientation. Table S1 summarizes the dimensions of the used for each system in the slit pore confinement geometry in terms of number of atoms, fluid thickness and the cell size in the direction parallel to the surface.

To achieve the appropriate bulk water density within the slit pore confinements, the piston methodology is implemented during the equilibration stage. The piston methodology entails maintaining the structure of the upper slab rigid and applying a per-atom force equivalent to a target pressure of 1 atm on the atoms of the uppermost layer. Equilibration is carried out until the upper slab oscillates around a constant z-value for a minimum of 200 ps. Subsequently, the position of the upper slab is fixed at the averaged position and all constraints are removed, except for the metal systems where the two bottom layers are kept fixed. Additional 500 ps of equilibration with constant height are carried out and the density profile along the z-direction is monitored. The configurations resulting from the equilibration runs are then used as initial structures for the final production runs. of 5.0 ns at the temperature of 330 K. A temperature of 330 K was selected instead of room temperature to compensate for the lower diffusivity of bulk water as predicted with the optB88-vdW functional (12) that the C-NNPs are based on.

3. Analysis of the Pt contact sub-layers

The density profiles of the water contact layer on the Pt(100) and Pt(111) surfaces, shown in Figures S10a and S10h, respectively, reveal a distinct separation of oxygen atoms into two sub-layers, each characterized by an individual density peak. The first sub-layer (denoted "ad1"), represented by the blue part in the density profile of Figure S10(a,h), exhibits a density peak in close proximity to the surface, at a distance of 2.25 Å. The second sub-layer (denoted "ad2"), indicated by the red part in the density profile of Figures S10(a,h), displays a density peak at a distance of 3.0 Å. Snapshots in Figure S10 [(b) and (c) for Pt(111), and (i) and (j) for Pt(100)] demonstrate that the oxygen atoms in the first sub-layer (blue) are localized at the top sites of individual Pt atoms with close contact, while the oxygen atoms in the second sub-layer (red) exhibit a more diffuse distribution.

The highly structured nature of the first sub-layer is evident in the 2D pair correlation function (PCF) shown in Figure S10(d,k), with a strong positional correlation among the oxygen atoms following the hexagonal arrangement of surface atoms on Pt(111) and the square lattice structure on Pt(100). On Pt(111), the 1D-PCF (inset of Figure S10d) reveals a highly anisotropic and inhomogeneous first sub-layer, as evidenced by a significant decrease in the density peak along both high symmetry axes, indicating a suppression of directly neighboring oxygen atoms. In contrast, the first sub-layer of Pt(100) exhibits inhomogeneity, with a significantly increased density peak along the [001] axis, promoting the proximity of neighboring oxygen atoms (inset of Figure S10k).

The 2D-PCF of the second sub-layer on Pt(111) (*cf.* Figure S10e) demonstrates a homogeneous and isotropic distribution of oxygen atoms, showing no influence from the highly ordered first sub-layer. Additionally the 1D-PCF (inset of Figure S10e) does not exhibit noticeable anisotropy. Conversely, on Pt(100), the 2D-PCF of the second sub-layer (*cf.* Figure S10m) displays significant anisotropy and inhomogeneity in the positional arrangement of oxygen atoms. The anisotropy in the first solvation shell along the high symmetry axes [011] and [001] result in a partially rectangular shape. Furthermore, the outer solvation shell exhibits inhomogeneity aligned with the square lattice structure, in addition to minor long-range positional correlations. Overall, the second sub-layer on Pt(100) demonstrates notable structural effects resulting from the highly ordered first sub-layer.

4. Hydrogen bond network at the interfaces

Figure S11 provides an analysis of the hydrogen bond network within the water slab on graphene, MoS_2, Au(111), Au(100), Pt(111), and Pt(100) interfaces, aiming to understand how the substrate and its induced structural features of the water contact layer effect the resulting hydrogen bond network. Hydrogen Bonds are defined following the geometrical criteria of Ref. (17) with a maximum distance between donor and acceptor up to $d_{\mathrm{O-H\cdots O}} \leq 3.2$Å and a angle $180^\circ \geq \theta_{\mathrm{O-H\cdots O}} \geq 130^\circ$. Further, only hydrogen bonds between water molecules are taken into account to neglect hydrogen accepting atoms of the substrate.

As described in Sec. *Microscopic structure of the water contact layer* the water contact layer on graphene exhibits no pronounced structural effects of the substrate due to rather low solid-liquid interactions. This unaffected contact layer results in the formation of a dense intra-layer hydrogen bond network in the thin contact layer (*cf.* Fig. S11a) lower left quadrant] with a similar share between donors and acceptors in this layer (*cf.* Fig. S11c). Further, a significant amount of bidirectional inter-layer bonds are formed between the contact layer and the preceding transition layer at $\Delta z \approx 0.6$nm, thus with both accepting and donating sides in each layer (*cf.* Fig. S11a upper left & lower right quadrant). As the following transition layer shows an increased density in comparison to the other studied interfaces, a comparable dense intra-layer hydrogen bond network results as well. Overall the amount of hydrogen bonds per water molecule is rather constant with 3.7 hydrogen bonds per water molecule as seen in Figure S11b. Only in close proximity to the surface it decreases due to some hydrogen atoms oriented towards the surface.

The water/MoS_2 interface reveals a hydrogen bond network similar to the graphene/water interface. In both cases, the donors and acceptors are at a similar distance from the surface, as can be seen in the lower left square in Figure S11d. This suggests a dense hydrogen bond network that extends parallel to the surface, as in graphene. Additionally, similarly to graphene, a well-balanced bond network intra-layer results, as can be seen by the equally pronounced peaks in the upper left and lower right quadrants.

The water contact layer on Au(111) exhibit only subtle influences of substrate induced structural effects, except an extension in direction to the substrate by slightly dragging individual water molecules closer to the surface due to increased solid-liquid interactions. This extension of the water contact layer results in a less dense parallel orientation of the hydrogen bond network, as it can be seen by the borader but less dense peak in the bottom left quadrant of Figure S11g. However, the intra-layer hydrogen bond network is still relatively uniform within the water contact layer as indicated by the single peak in the lower left

quadrant in Figure S11g. The water molecules spreading towards the surface predominantly donate their bound hydrogen atoms for hydrogen bonds towards the contact layer as seen by the increasing amount of donor atoms in Figure S11i. As the density in the transition layer gets less dense, on the metal interfaces the density of hydrogen network decreases accordingly. However, as seen in the upper left, respectively lower right quadrant of Figure S11g there are still significant amounts of inter-layer hydrogen bonds between the transition- and contact layer, with a slightly increased amount of donating bonds from the transition to the contact layer.

The water contact layer on Au(100) shows a rather broad distribution with the lowest density peak with increased substrate induced structural effects as shown in Sec. *Microscopic structure of the water contact layer*. Thus, the contact layer shows a rather loose hydrogen bond network with a diffuse distribution along the contact layer as seen by the broad distribution in the lower left quadrant in Figure S11j. Additionally the inter-layer bonding is reduced between the layers as well as the intra-layer hydrogen bond network within the transition layer. (*cf.* upper left/lower right, respectively upper right quadrant in Figure S11j). Similar to the Au(111) interface water molecules in close proximity to the surface predominantly donate their bound hydrogen atoms for hydrogen bonds towards the contact layer as seen in Figure S11j.

On Pt(111), shown in Figure S11m, there is a clear splitting of the water hydrogen bond network into individual domains within the contact layer. These domains correspond to the distinct feature of the Pt interfaces of the split contact layer into two sub-layers as described previously in Sec. S3. In accordance to the described suppressing of direct neighboring water molecules in the first sub-layer no hydrogen bonds exists between neighboring water molecules in the first sub-layer. Consequently these water molecules acting as donors towards the overlaying sub-layer "ad2". These intra-layer bonds are highly unidirectional with only minor donating hydrogen bonds from the second sub-layer towards the first sub-layer. Similar to Au(111), an inter-layer network is formed between the contact layer and the transition layer, with bridging bonds from the transition layer slightly predominating in the direction of the contact layer (*cf.* upper left/lower right in Figure S11j).

On Pt(100), a very similar hydrogen bond network results as on Pt(111), but now with hydrogen bonding occurring between the near-surface water molecules in the first sub-layer, as evident by the peak at $\Delta z_{\text{acceptor}} \approx \Delta z_{\text{donor}} \approx 0.225\,\text{nm}$ in Figure S11p. This also enables the formation of hydrogen bonds donating from the second sub-layer towards the first sub-layer. However, the majority of the hydrogen bonds is still donated from the first- to the second sub-layer as seen in the lower left quadrant in Figure S11p,r.

5. Fluctuations in the instantaneous dividing interface

While in the main part of the manuscript in Sec. *Hydrophobes' adsorption and connection to wetting* the fluctuations of the instantaneous dividing interface (h_{inst} in Figure S12a) are investigated, here we give additional insights into the fluctuations of the instantaneous solid surfaces and the fluctuations of the instantaneous dividing interface on planar fixed graphene.

An analysis of the height fluctuations of the instantaneous solid surface, represented by the red dotted line in Figure S12b, revealed significant oscillations of the graphene layer around its average position due to its inherent flexibility as a 2D material. The water interface instantly adapts to these fluctuations of the solid surface, leading to an increase in its own fluctuations relative to the average surface position (red dotted line in Figure S12b). This is confirmed by fixing the graphene layer, which leads to distributions (*cf.* orange solid line in Fig. S12b) almost identical to the fluctuations relative to the instantaneous surface in the case of flexible graphene *cf.* red solid line in Fig. S12b). Interestingly, slightly increased fluctuations are observed in the graphene sheet itself when it is in contact with water (red half-dashed line in Fig. S12b) compared to its stand-alone fluctuations in vacuum, which is shown by the blue half-dashed line in Fig. S12b.

The stiffer nature of the metal interfaces is evident from the narrow distributions of their fluctuations around their average positions, as shown in Figure S12c. While both gold and platinum are rigid compared to graphene, platinum shows less fluctuating behavior due to increased interactions between the solid atoms. Furthermore, for both materials the fluctuations on the less dense square lattice (100) surfaces are increased compared to their hexagonal terminated (111) equivalents.

6. Dependence of the water contact angle on the size of the hydrophobe

The determination of the water contact angle using the free excess solvation energy $\delta\mu$ from the adsorption of hydrophobic particles is analogous to the method presented in Godawat et al. (18). The contact angle can be determined by the minimum of the free excess solvation energy of a hydrophobic particle with the radius R. As shown in Figure S15, the contact angle as a function of particle radii converges for $R \geq 2.75,\text{Å}$ for all substrates, except for Au(111) and Au(100). As such we choose $R \geq 2.75,\text{Å}$ to provide an estimate of the contact angle. Although we cannot determine the contact angle on Au(111) and Au(100) with high accuracy, our results indicate that graphene and MoS_2 present similar wetting behaviour at the boundary between hydrophilicity and hydrophobicity, that both gold surfaces are hydrophilic and that Au(111) is more wetting than Au(100), and that the platinum surfaces are completely wetting.

Table S1. Dimensions of confinements used in final production runs. L_x and L_y denote the lateral dimension of the simulation box, h_{slit} is the distance between the substrate surfaces and n_{atoms} denotes the total number of atoms in the system, i.e. water and substrate.

System	L_x (nm)	L_y (nm)	h_{slit} (nm)	n_{atoms}
Graphene	2.98620	3.20187	6.78	7568
MoS_2	3.15	3.2736	6.94	8370
Au(111)	3.51714	3.04594	6.78	8862
Au(100)	3.51714	3.51714	6.80	10056
Pt(111)	3.36124	2.91092	7.15	8202
Pt(100)	3.36124	3.36124	7.05	9282

Table S2. The friction coefficients $\lambda_{\rm fit}$ obtained by using the double exponential model in Ref. (19) and $\lambda_{\rm max}$, obtained from the maximum in $\Lambda(t)$ for each aqueous interface system, along with the resulting slip-length b which was extracted from $\lambda_{\rm fit}$.

System	$\lambda_{\rm fit}$ $(10^4$ Ns/m$^3)$	$\lambda_{\rm max}$ $(10^4$ Ns/m$^3)$	b (nm)
Graphene	5.36	5.85 ± 0.17	18.12
MoS_2	53.54	48.51 ± 1.13	1.87
Au(111)	31.31	31.59 ± 1.15	3.19
Au(100)	80.03	75.93 ± 2.27	1.25
Pt(111)	127.18	120.69 ± 2.21	0.79
Pt(100)	221.51	197.32 ± 2.36	0.45

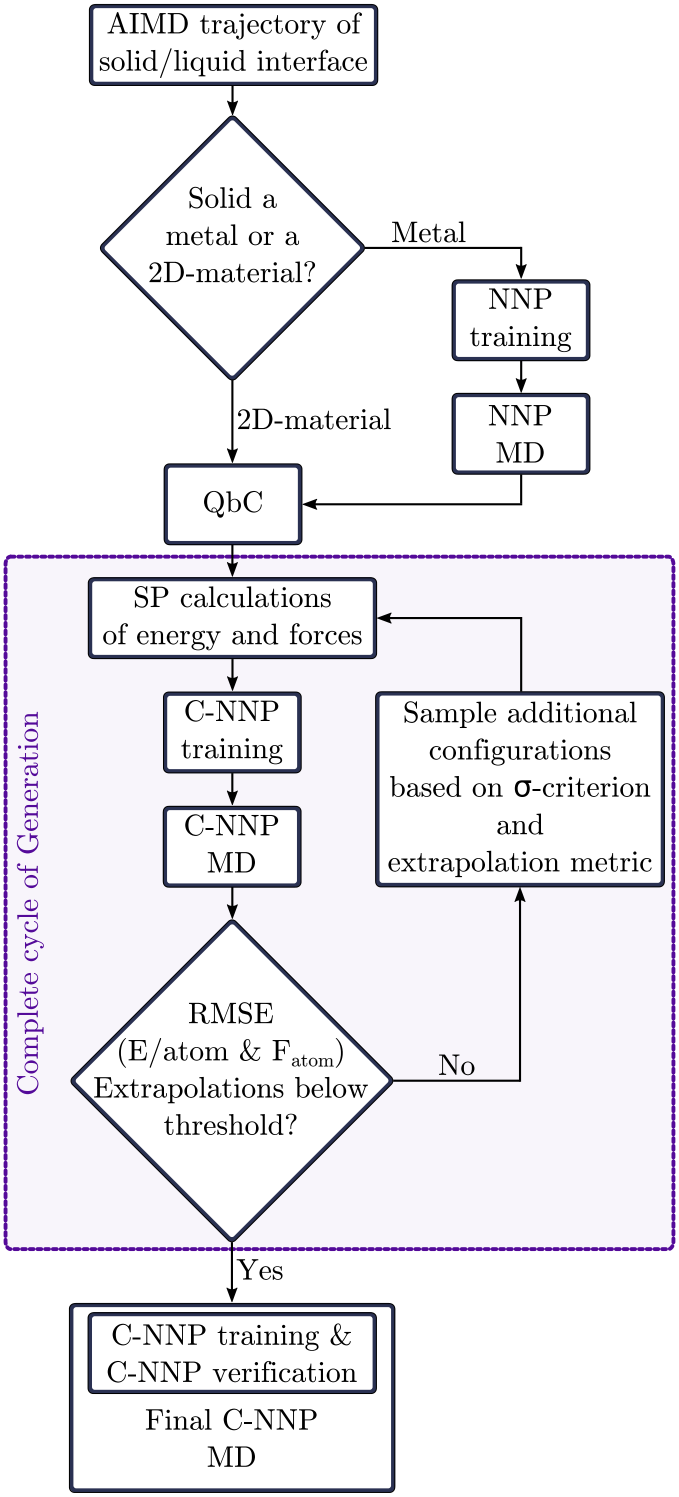

Fig. S1. Schematic representation of the iterative workflow to parameterize an individual Committee Neural Network Potential.

Generations of the C-NNP models for the aqueous graphene and for the aqueous MoS_2 interfaces

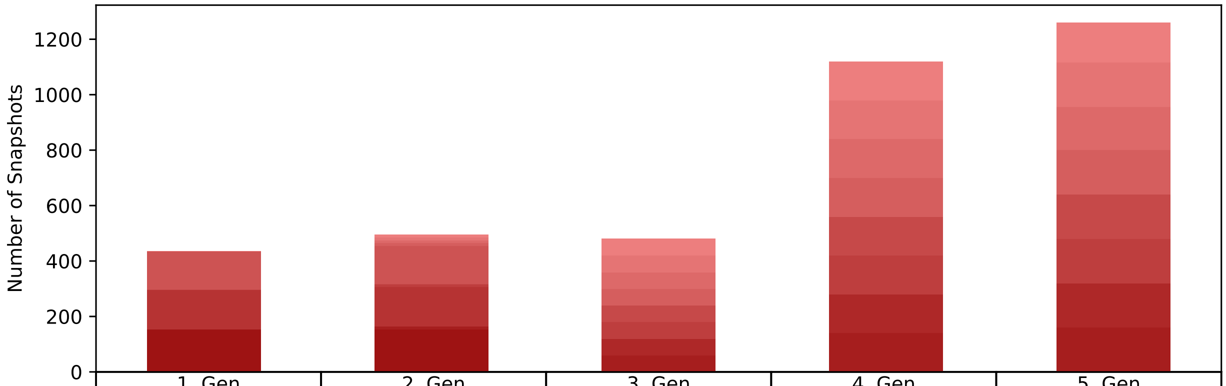

	1. Gen	2. Gen	3. Gen	4. Gen	5. Gen
Gra-0.9nm-5x3	154	154 (0)	0 (-154)	0 (0)	0 (0)
Gra-0.9nm-10x6	0	10 (+10)	60 (+50)	140 (+80)	160 (+20)
Gra-1.2nm-10x6	0	0 (0)	60 (+60)	140 (+80)	160 (+20)
Gra-1.5nm-5x3	143	143 (0)	0 (-143)	0 (0)	0 (0)
Gra-1.5nm-10x6	0	10 (+10)	60 (+50)	140 (+80)	160 (+20)
Gra-1.7nm-10x6	0	0 (0)	60 (+60)	140 (+80)	160 (+20)
Gra-2.0nm-5x3	138	138 (0)	0 (-138)	0 (0)	0 (0)
Gra-2.0nm-10x6	0	10 (+10)	60 (+50)	140 (+80)	160 (+20)
Gra-2.5nm-10x6	0	10 (+10)	60 (+50)	140 (+80)	156 (+16)
Gra-10x6$_{Surf}$	0	10 (+10)	60 (+50)	140 (+80)	160 (+20)
Bulk water	0	10 (+10)	60 (+50)	140 (+80)	144 (+4)
Total	435	495 (+60)	480 (-15)	1120 (+640)	1260 (+140)

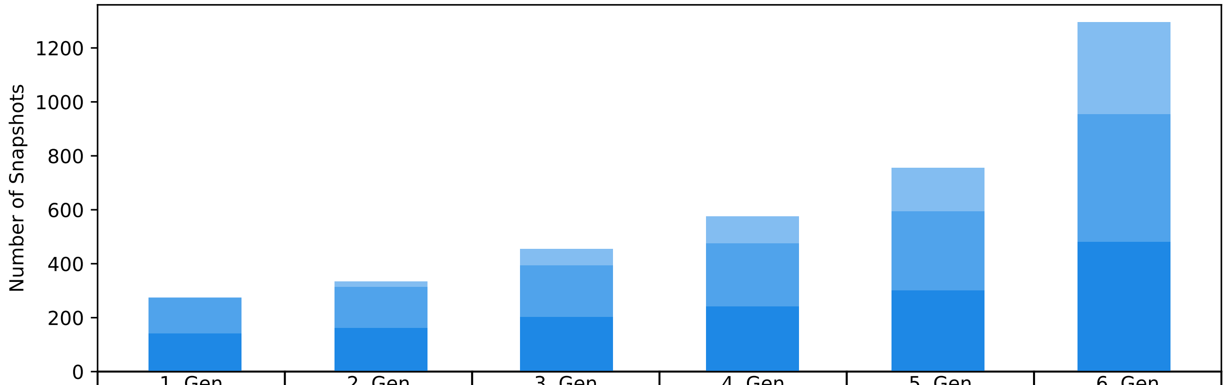

	1. Gen	2. Gen	3. Gen	4. Gen	5. Gen	6. Gen
MoS_2-2.7nm-8x4	142	162 (+20)	202 (+40)	242 (+40)	302 (+60)	482 (+180)
MoS_2-8x4$_{Surf}$	133	153 (+20)	193 (+40)	233 (+40)	293 (+60)	473 (+180)
Bulk water	0	20 (+20)	60 (+40)	100 (+40)	160 (+60)	340 (+180)
Total	275	335 (+60)	455 (+120)	575 (+120)	755 (+180)	1295 (+540)

Fig. S2. Evolution of the size and systems used in the training sets for the water/graphene and water/MoS_2 committee neural network potentials (C-NNPs).

Generations of the C-NNP models for the aqueous Platinum and for the aqueous Gold interfaces

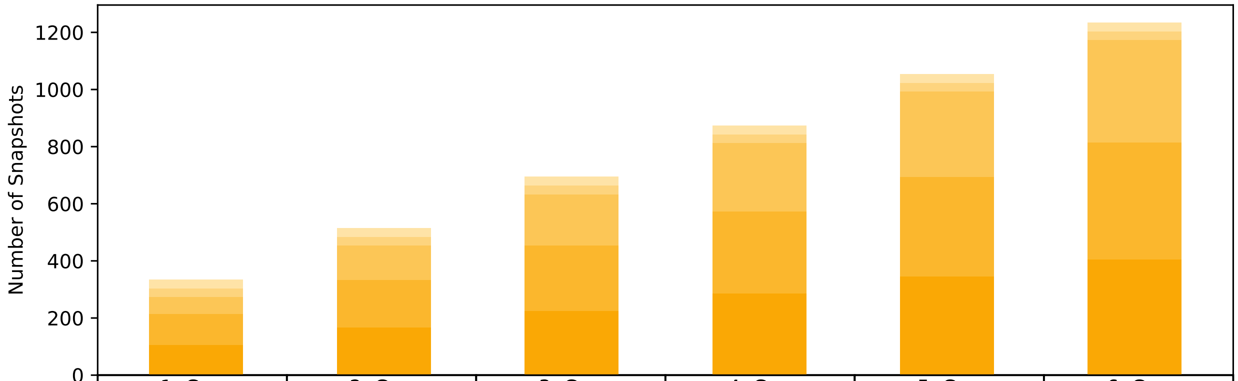

	1. Gen	2. Gen	3. Gen	4. Gen	5. Gen	6. Gen
Au(100)-8x8	106	166 (+60)	226 (+60)	286 (+60)	346 (+60)	406 (+60)
Au(111)-8x8	108	168 (+60)	228 (+60)	288 (+60)	348 (+60)	408 (+60)
Bulk water	60	120 (+60)	180 (+60)	240 (+60)	300 (+60)	360 (+60)
Au(100)-8x8$_{Surf}$	30	30 (+0)	30 (+0)	30 (+0)	30 (+0)	30 (+0)
Au(111)-8x8$_{Surf}$	30	30 (+0)	30 (+0)	30 (+0)	30 (+0)	30 (+0)
Total	334	514 (+180)	694 (+180)	874 (+180)	1054 (+180)	1234 (+180)

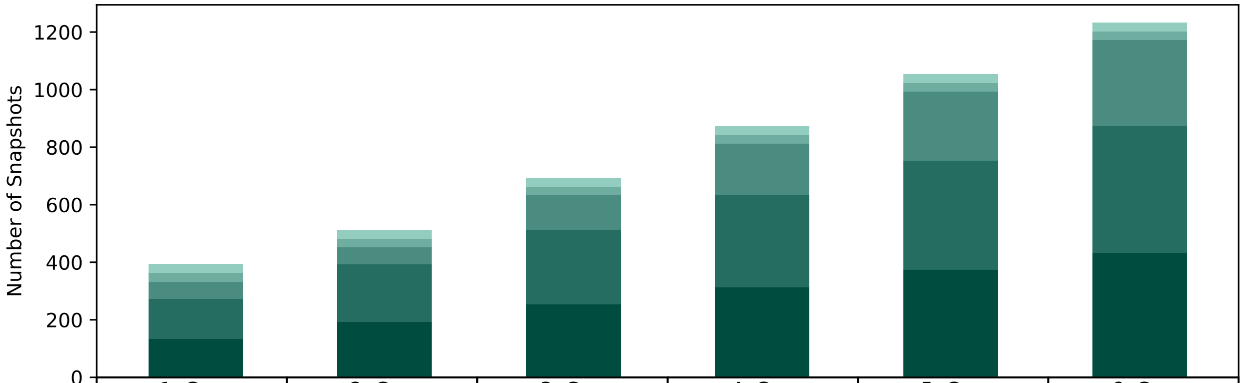

	1. Gen	2. Gen	3. Gen	4. Gen	5. Gen	6. Gen
Pt(100)-8x8	134	194 (+60)	254 (+60)	314 (+60)	374 (+60)	434 (+60)
Pt(111)-8x8	139	199 (+60)	259 (+60)	319 (+60)	379 (+60)	439 (+60)
Bulk water	60	60 (+0)	120 (+60)	180 (+60)	240 (+60)	300 (+60)
Pt(100)-8x8$_{Surf}$	30	30 (+0)	30 (+0)	30 (+0)	30 (+0)	30 (+0)
Pt(111)-8x8$_{Surf}$	30	30 (+0)	30 (+0)	30 (+0)	30 (+0)	30 (+0)
Total	393	513 (+120)	693 (+180)	873 (+180)	1053 (+180)	1233 (+180)

Fig. S3. Evolution of the size and systems used in the training sets for the water/gold and water/platinum C-NNPs.

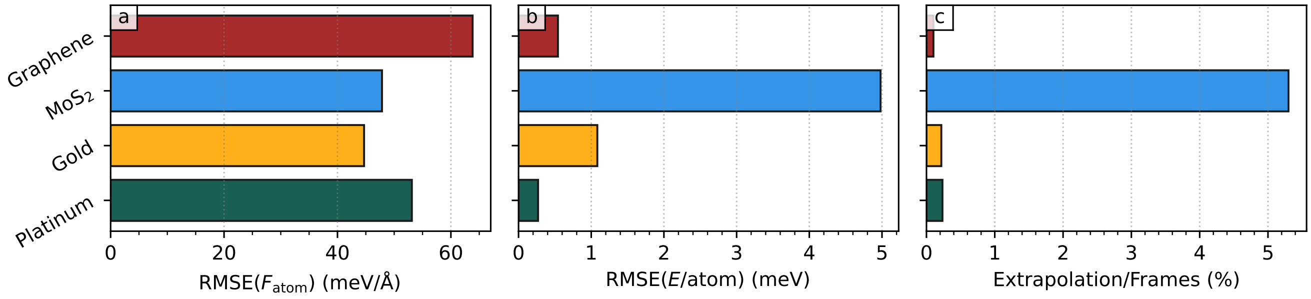

Fig. S4. Metrics for the final Generation of C-NNPs for graphene, MoS_2, Gold and Platinum. (a) RMSE of the atomic forces and (b) RMSE of the total energy per atom between *ab initio* and C-NNP calculations for 60 random sampled configurations for each system. (c) Ratio of frames causing a extrapolation in any ACSF during the final 500 ps.

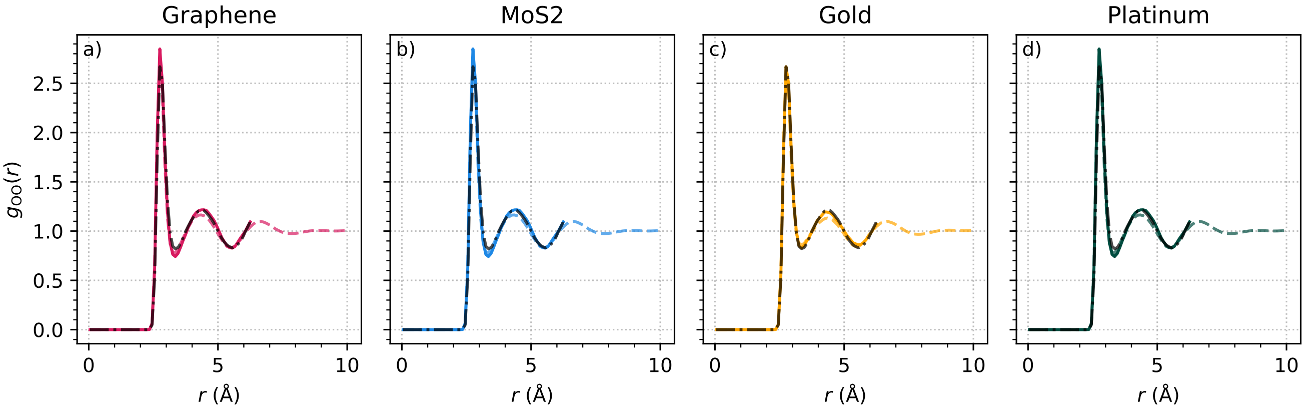

Fig. S5. Oxygen-oxygen radial distribution function of bulk water using C-NNP MD calculations of 32 molecules (full lines) and 256 molecules (dotted lines) based on the a) graphene, b) MoS_2, c) Au and d) Pt model in comparison to optB88-vdW AIMD (black dotted line) calculations of 32 molecules from Ref. (12).

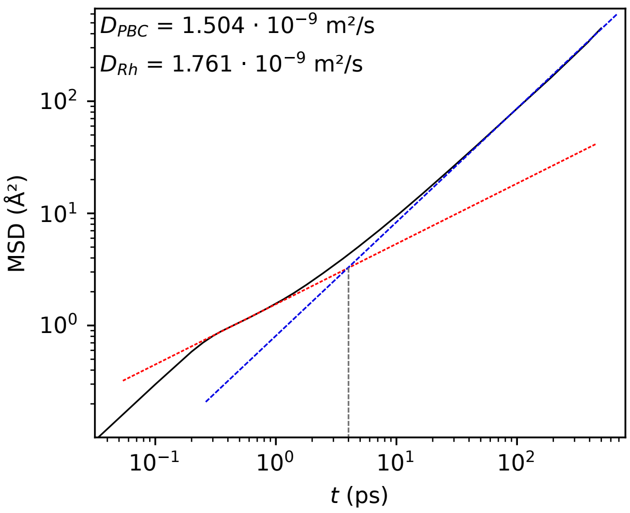

Fig. S6. Example for the extraction of the MSD of bulk water for a 500 ps trajectory obtained with the water/Platinum interface C-NNP model. The transition from the ballistic regime (red dotted line) to the diffusive regime (blue dotted line) is estimated by the intersection of two linear regressions.

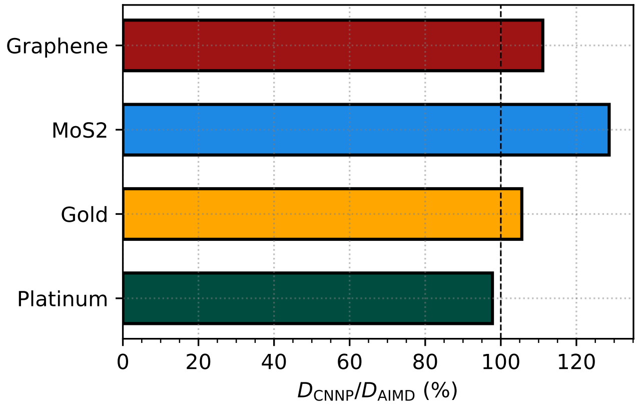

Fig. S7. Diffusion coefficients of water in the bulk phase obtained from each individual C-NNP model in relation to AIMD results computed with the same functional used in the training (optB88-vdW) and at 330 K from Ref.(12).

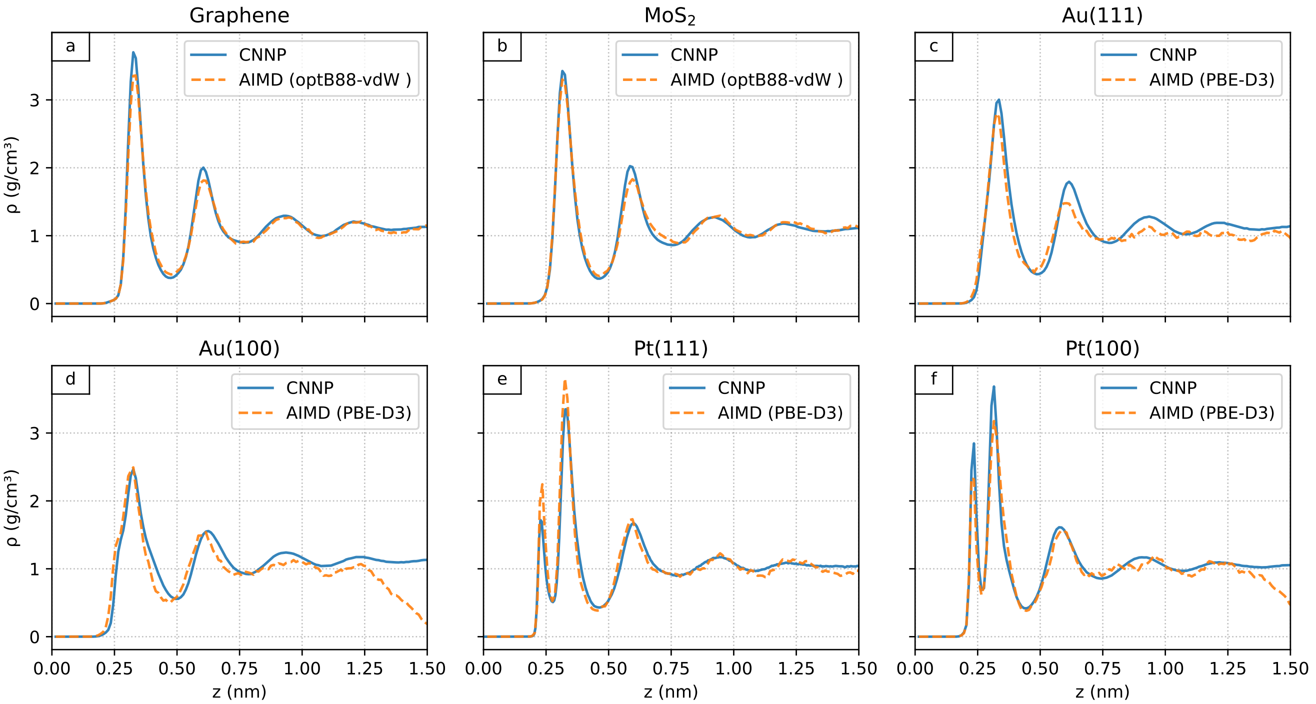

Fig. S8. Water density profiles on studied substrates: (a) graphene, (b) MoS_2, (c) Au(111), (d) Au(100), (e) Pt(111), and (f) Pt(100). Profiles derived from C-NNPs (blue) and *ab-initio* (orange) methods.

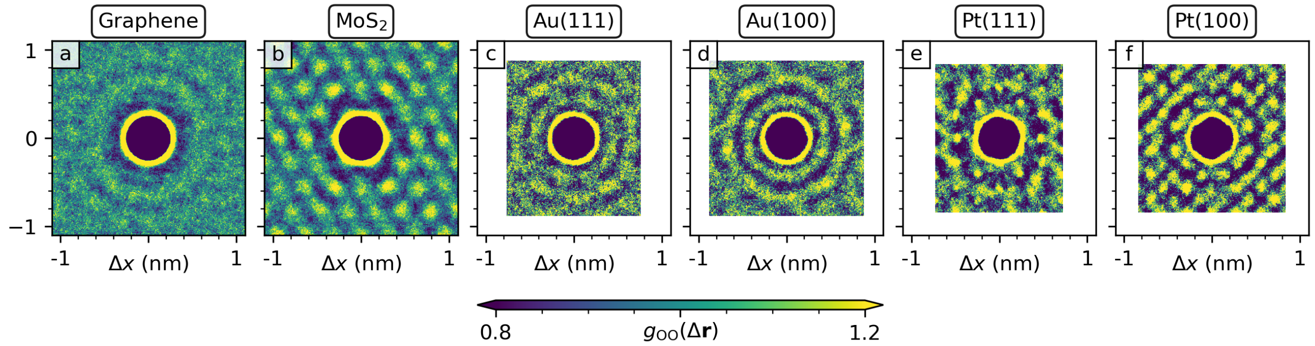

Fig. S9. Two-dimensional oxygen-oxygen pair correlation of the considered systems obtained from AIMD calculations. Data for graphene (a) and MoS_2 (b) are from Ref. (7) using the optb88-VdW functional. Au(111) (c), Au(100) (d), Pt(111) (e), and Pt(100) (f) using the PBE-D3 functional.

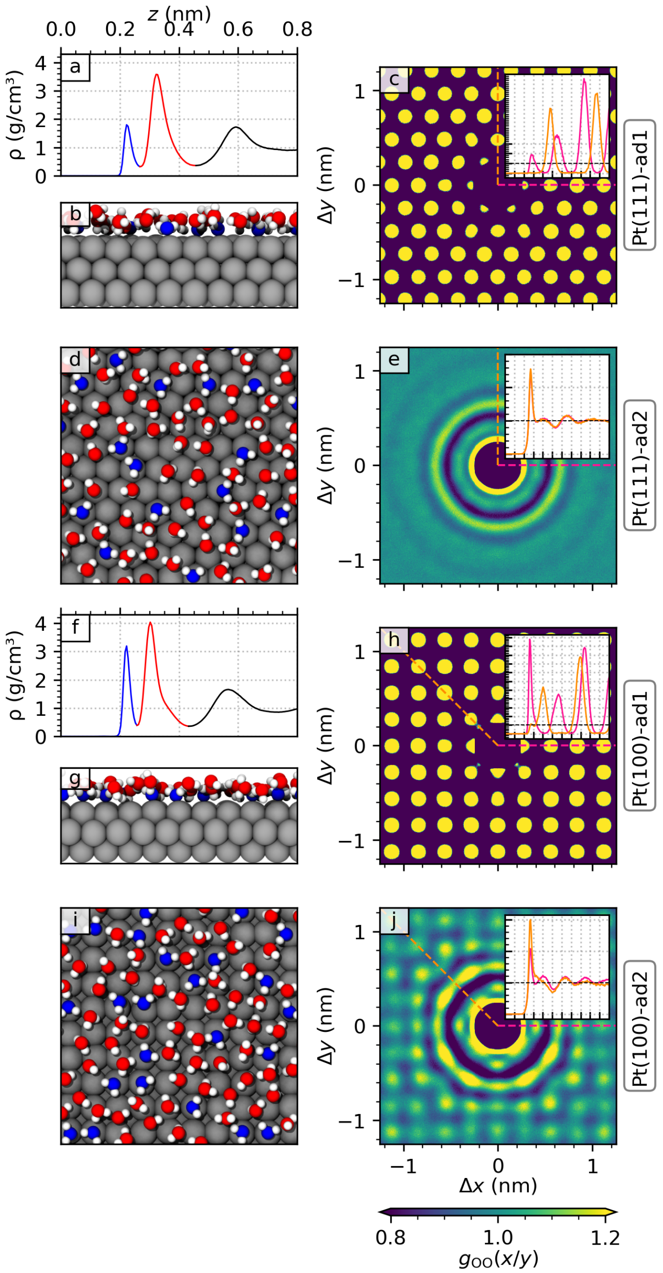

Fig. S10. Water density profiles on (a) the Pt(111) surface and (f) the Pt(100) surface. The area of the first "ad1" and second "ad2" sub-layer are marked in blue and red, respectively. The same coloring scheme is used to mark the oxygen atoms in the side views (b,g) and top views (d,i) of the respective surface according to their position either in the first or second sub-layer. In-plane structure and intermolecular correlations in first sub-layer (c) and second sub-layer (e) on Pt(111), respectively. (h) and (j) showing the same data for the Pt(100) surface. Insets showing the one dimensional representation along the two corresponding surface specific high symmetry axes.

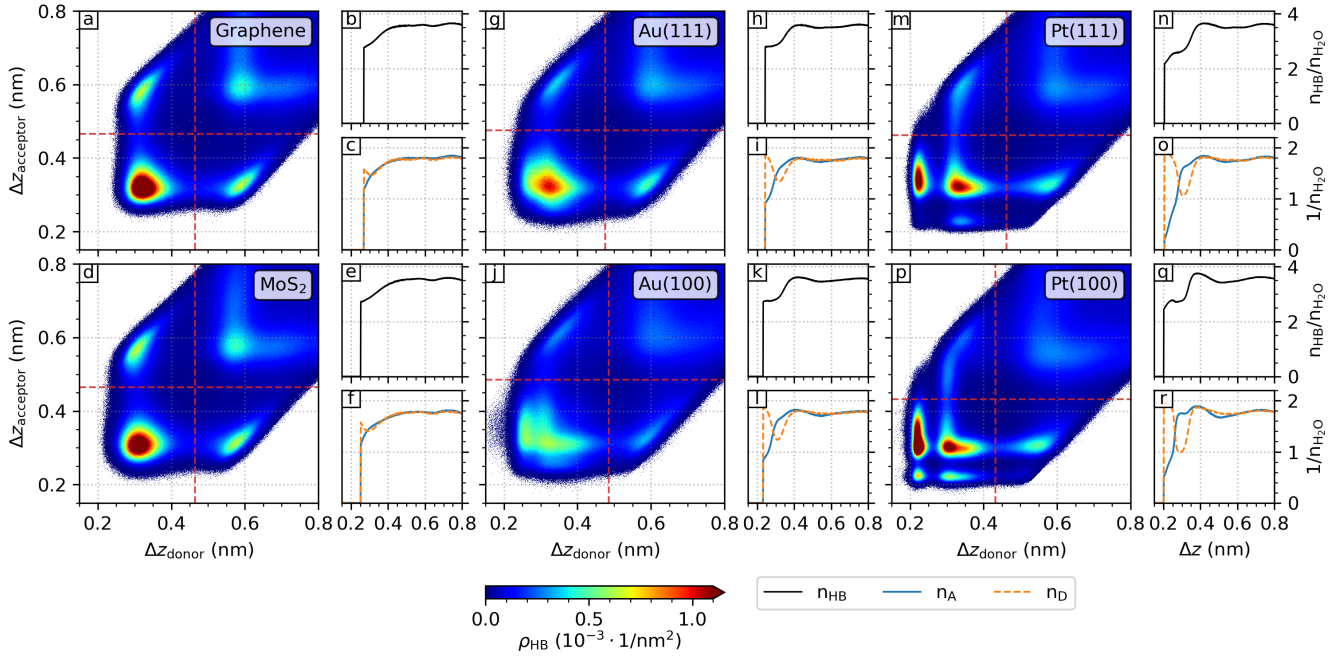

Fig. S11. Hydrogen bond network at the water-solid interface of graphene, MoS_2, Au(111), Au(100), Pt(111), and Pt(100). Contour plots (a, d, g, j, m, p) show hydrogen bond density as a function of the donor's and acceptor's distance to the surface (Δz). Dotted red lines mark the contact layer boundaries. Resulting quadrants represent bonds in-between the contact layer (lower left), from the transition layer to the contact layer (lower right), from the contact layer to the transition layer (upper left), and in-between the transition layer (upper left). The one-dimensional plots (b, e, h, k, n, q) show the total number hydrogen bonds per water molecule as a function of height, while (c, f, i, l, o, r) show the number of donating and accepting bonds per water molecule as a function of height.

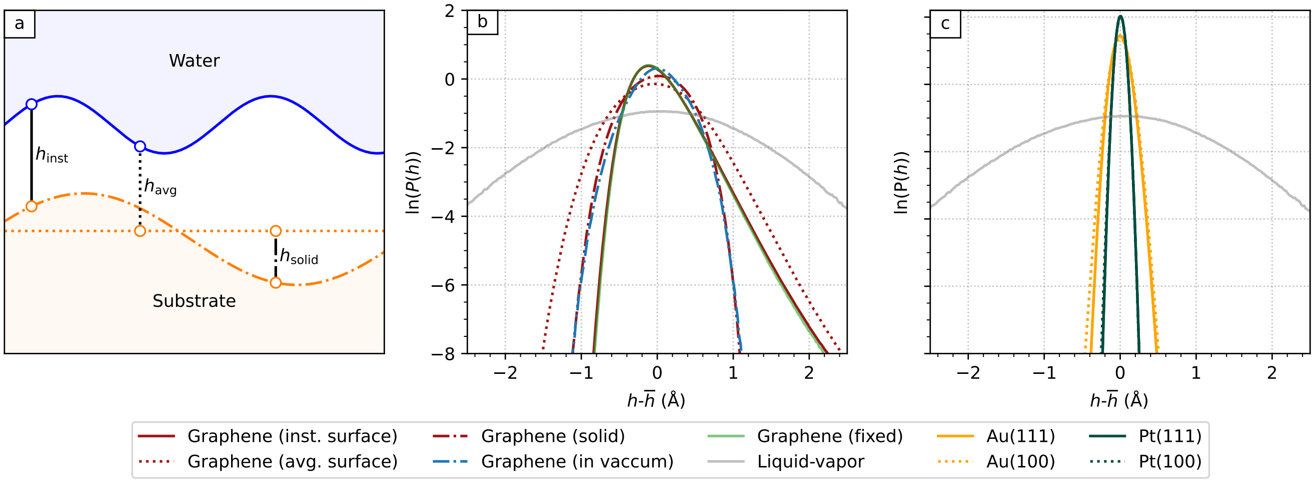

Fig. S12. (a) Schematic representation of the different height references used to calculate fluctuations in the instantaneous interface and solid surface. (b) Extended analysis of the probability distributions of the height fluctuations of graphene and its water interface. The red colored plots represent the presented case of a fully flexible graphene surface in contact with water. The blue half-dashed line shows the fluctuations of the graphene surface around its average position in vacuum, while the orange curve shows the fluctuations of the instantaneous water interface in contact with a fixed planar graphene surface. (c) Probability distributions of the height fluctuations of the metal interfaces around their average height.

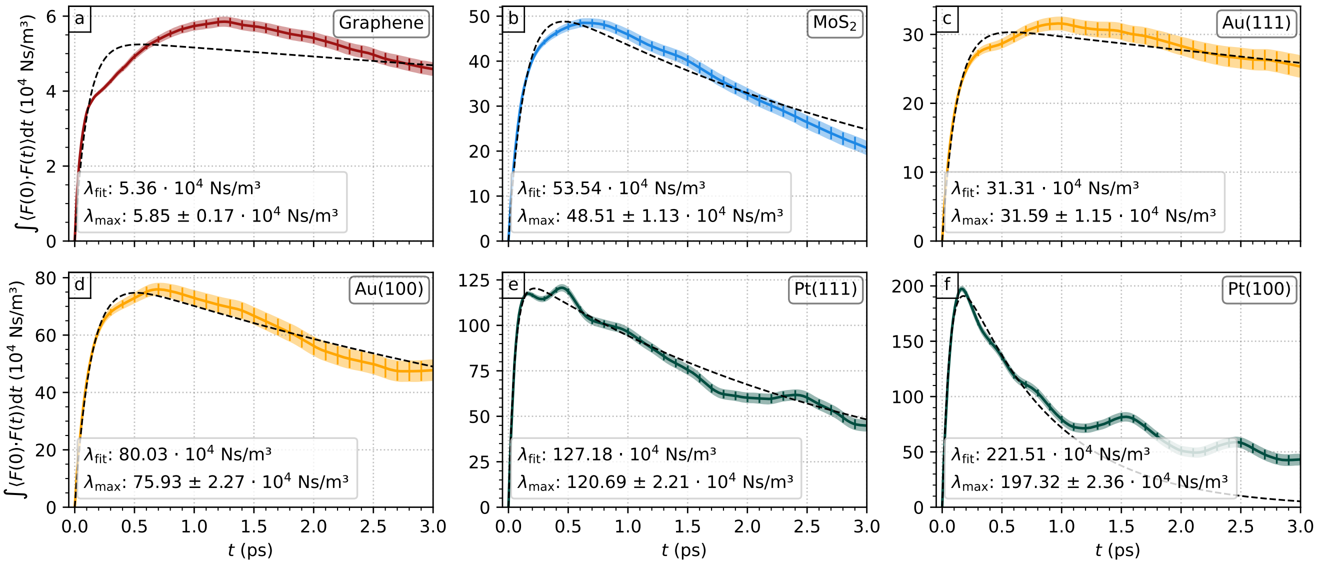

Fig. S13. Green-Kubo Integral of the force auto correlation function on graphene, MoS_2, Au(100), Au(111), Pt$(100)$and Pt(111). The black dotted line shows the fit of the expression from Ref. 19 for $\Lambda(t)$. λ_{fit} refers to the value of the friction coefficient in the long-time limit resulting from this fit, and λ_{max} is the value the one obtained from the maximum in the GK curve as done in Refs. (7, 19). Note that despite some deviations from the fit for graphene and Pt)(100), the difference between λ_{max} and λ_{fit} is of about 10 %, and such deviations have a negligible impact on the results related to slippage and diffusio-osmotic transport. Nevertheless, we speculate that for graphene deviations may arise because the model in Ref. (19) do not account for the possible role of the height fluctuations of the sheet while for Pt(100) it may be because they do not account for the sluggish dynamics associated to the strong chemisorption on this surface.

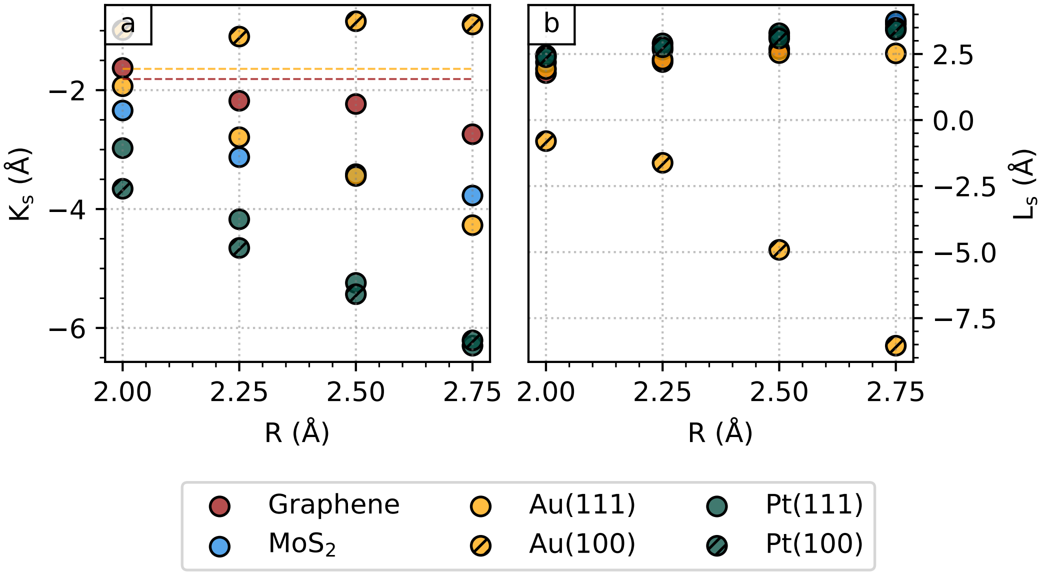

Fig. S14. Length scale characteristics of hydrophobe particles $\mathrm{K_s}$ (a) and $\mathrm{L_s}$ (b) at the aqueous interfaces of graphene, MoS_2, Au(111), Au(100), Pt(111) and Pt(100). The waters length scale characteristic $\mathrm{K_w}$ are shown with dotted lines of the respective color for the interfaces which exhibit a sign change in the term of $\mathrm{K_s} - \mathrm{K_w}$ for changing radii R, namely graphene and Au(111).

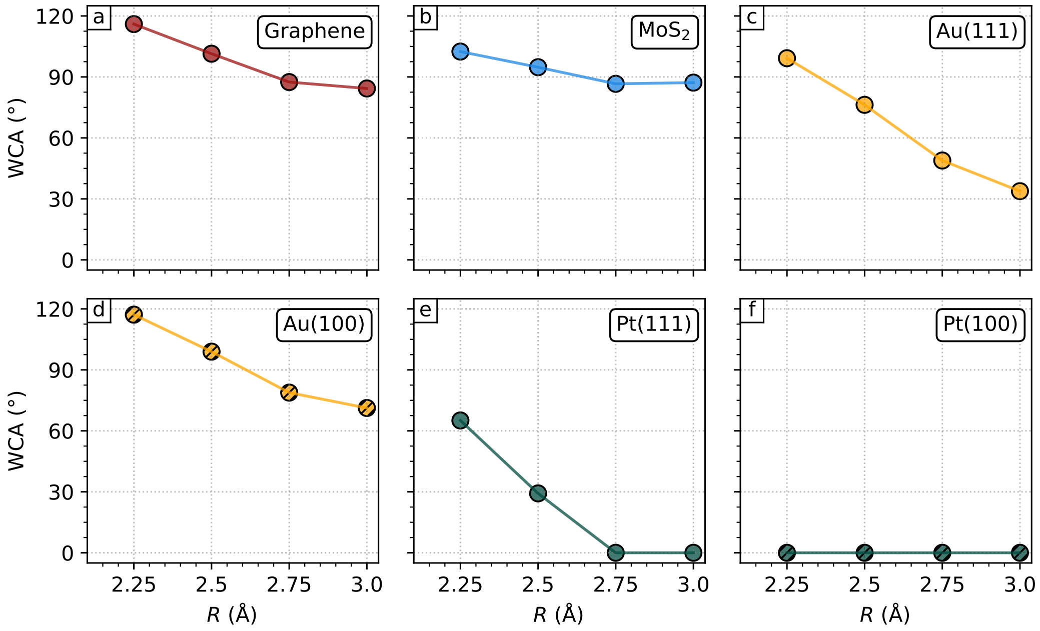

Fig. S15. Dependence of the water contact angle (WCA) on the size of the hydrophobe denoted by the radius R using Eq. 6 in the main text for the each substrate consisting of (a) graphene, (b) MoS_2, (c) Au(111), (d) Au(100), (e) Pt(111), and (f) Pt(100).

References

1. C Schran, et al., Machine learning potentials for complex aqueous systems made simple. *Proc. Natl. Acad. Sci.* **118**, e2110077118 (2021).
2. C Schran, K Brezina, O Marsalek, Committee neural network potentials control generalization errors and enable active learning. *The J. Chem. Phys.* **153**, 104105 (2020).
3. A Singraber, T Morawietz, J Behler, C Dellago, Parallel multistream training of high-dimensional neural network potentials. *J. Chem. Theory Comput.* **15**, 3075–3092 (2019).
4. J Gäding, G Tocci, Supporting Data for the Paper: The role of the water contact layer on hydration and transport at solid/liquid interface. *Zenodo, https://doi.org/10.5281/zenodo.12774464* (2024).
5. J Behler, Constructing high-dimensional neural network potentials: A tutorial review. *Int. J. Quantum Chem.* **115**, 1032–1050 (2015).
6. M Eckhoff, J Behler, High-dimensional neural network potentials for magnetic systems using spin-dependent atom-centered symmetry functions. *npj Comput. Mater.* **7** (2021).
7. G Tocci, M Bilichenko, L Joly, M Iannuzzi, Ab initio nanofluidics: disentangling the role of the energy landscape and of density correlations on liquid/solid friction. *Nanoscale* **12**, 10994–11000 (2020).
8. G Tocci, Ab initio molecular dynamics trajectories of liquid water at the interface with graphene sheets. *Zenodo, https://doi.org/10.5281/zenodo.6828034* (2022).
9. G Tocci, Ab initio molecular dynamics trajectories of liquid water at the interface with MoS2 sheets. *Zenodo, https://doi.org/10.5281/zenodo.6828539* (2022).
10. TD Kühne, Second generation Car–Parrinello molecular dynamics. *WIREs Comput. Mol. Sci, 2014* **4**, 391–406 (2014).
11. J Lan, VV Rybkin, M Iannuzzi, Ionization of water as an effect of quantum delocalization at aqueous electrode interfaces. *The J. Phys. Chem. Lett.* **11**, 3724–3730 (2020).
12. C Herrero, M Pauletti, G Tocci, M Iannuzzi, L Joly, Connection between water's dynamical and structural properties: Insights from ab initio simulations. *Proc. Natl. Acad. Sci.* **119**, e2121641119 (2022).
13. IC Yeh, G Hummer, System-size dependence of diffusion coefficients and viscosities from molecular dynamics simulations with periodic boundary conditions. *The J. Phys. Chem. B* **108**, 15873–15879 (2004).
14. J Behler, Four generations of high-dimensional neural network potentials. *Chem. Rev.* **121**, 10037–10072 (2021) PMID: 33779150.
15. J Behler, M Parrinello, Generalized neural-network representation of high-dimensional potential-energy surfaces. *Phys. Rev. Lett.* **98**, 146401 (2007).
16. AP Thompson, et al., LAMMPS - a flexible simulation tool for particle-based materials modeling at the atomic, meso, and continuum scales. *Comput. Phys. Commun.* **271**, 108171 (2022).
17. GA Jeffrey, GA Jeffrey, *An introduction to hydrogen bonding.* (Oxford university press New York) Vol. 12, (1997).
18. R Godawat, SN Jamadagni, S Garde, Characterizing hydrophobicity of interfaces by using cavity formation, solute binding, and water correlations. *Proc. Natl. Acad. Sci.* **106**, 15119–15124 (2009).
19. H Oga, Y Yamaguchi, T Omori, S Merabia, L Joly, Green-kubo measurement of liquid-solid friction in finite-size systems. *The J. Chem. Phys.* **151**, 054502 (2019).
